# FACT regulates pluripotency through proximal and distal regulation of gene expression in murine embryonic stem cells

**DOI:** 10.1186/s12915-023-01669-0

**Published:** 2023-08-04

**Authors:** David C. Klein, Santana M. Lardo, Kurtis N. McCannell, Sarah J. Hainer

**Affiliations:** 1 Department of Biological Sciences, University of Pittsburgh, Pittsburgh, PA 15213 USA; 2 Department of Biology and Epigenetics Institute, University of Pennsylvania, Philadelphia, PA 19104 USA; 3 UPMC Hillman Cancer Center, University of Pittsburgh, Pittsburgh, PA USA

**Keywords:** FACT, Chromatin, Histone chaperone, Transcription, Embryonic stem cells, Pluripotency, Nucleosome, RNA, Histones, Genomics

## Abstract

**Background:**

The FACT complex is a conserved histone chaperone with critical roles in transcription and histone deposition. FACT is essential in pluripotent and cancer cells, but otherwise dispensable for most mammalian cell types. FACT deletion or inhibition can block induction of pluripotent stem cells, yet the mechanism through which FACT regulates cell fate decisions remains unclear.

**Results:**

To explore the mechanism for FACT function, we generated AID-tagged murine embryonic cell lines for FACT subunit SPT16 and paired depletion with nascent transcription and chromatin accessibility analyses. We also analyzed SPT16 occupancy using CUT&RUN and found that SPT16 localizes to both promoter and enhancer elements, with a strong overlap in binding with OCT4, SOX2, and NANOG. Over a timecourse of SPT16 depletion, nucleosomes invade new loci, including promoters, regions bound by SPT16, OCT4, SOX2, and NANOG, and TSS-distal DNaseI hypersensitive sites. Simultaneously, transcription of *Pou5f1* (encoding OCT4), *Sox2*, *Nanog*, and enhancer RNAs produced from these genes’ associated enhancers are downregulated.

**Conclusions:**

We propose that FACT maintains cellular pluripotency through a precise nucleosome-based regulatory mechanism for appropriate expression of both coding and non-coding transcripts associated with pluripotency.

**Supplementary Information:**

The online version contains supplementary material available at 10.1186/s12915-023-01669-0.

## Background

The process of transcription, or generation of RNA from a DNA template, is essential to all life and highly regulated at all stages (reviewed in [1–4]). A major barrier to transcription by RNA Polymerase II (RNAPII) is the presence of assembled nucleosomes occluding access to the DNA template (reviewed in [5–9]). Chromatin is highly dynamic and carefully regulated to promote or repress expression of certain genes as dictated by cell signaling, environmental conditions, and master regulators of cell fate. Nucleosomes can be altered through inclusion of histone variants and/or histone modifications (reviewed in [10–12]). Histone modifications are epigenetic post-translational marks that facilitate or prevent recruitment of proteins and signify particular chromatin states; for example, trimethylation of histone H3 at lysine residue 4 (H3K4me3) is found at regions of active transcription, while acetylation of histone H3 at lysine 27 (H3K27ac) identifies canonical active enhancers, with enrichment at promoters as well (reviewed in [11, 13, 14]).

In addition to histone variants and histone modifications, many proteins can regulate chromatin structure. Nucleosome remodeling factors translocate DNA using ATP hydrolysis to permit mobilization of nucleosomes and histone chaperones are responsible for adding and removing histone components, including both core histones and their variant substitutes, without hydrolyzing ATP (reviewed in [5, 15–18]). To create an RNA product, RNAPII coordinates with many proteins, including histone chaperones to overcome the physical hindrance of nucleosome-compacted DNA (reviewed in [6, 19–22]). RNAPII can facilitate this nucleosome disassembly [23], but the polymerase is often assisted by various histone chaperones that can facilitate removal of H2A/H2B dimers (as well as other combinations of histone proteins) and subsequent reassembly after the polymerase has passed [24–27]. One prominent histone chaperone is the FAcilitates Chromatin Transcription or FAcilitates Chromatin Transactions (FACT) complex.

The mammalian FACT complex is a heterodimer composed of a dimer exchange subunit, Suppressor of Ty 16 homolog (SUPT16H or SPT16) and an HMG-containing subunit that facilitates localization and DNA binding, Structure-Specific Recognition Protein 1 (SSRP1) [26, 28–30]. In *S. cerevisiae*, Spt16 forms a complex with Pob3, assisted by Nhp6, which has been proposed to fulfill the roles of the SSRP1 HMG domain [29–34]. In vitro, addition of FACT facilitates RNAPII passage through the nucleosomal roadblock—both disassembling nucleosomes prior to polymerase passage and repairing the nucleosome in the wake of the polymerase [19, 28–30, 35–39]. This role is consistent with the interaction between FACT and replication machinery [21, 37, 40, 41]. Given these dual roles in transcription and DNA replication, FACT has been thought to be crucial for cell growth and proliferation [29, 32, 35, 40–43]. More recent data has shown that while FACT is not required for cell growth in most healthy adult cell types, FACT is highly involved in cancer-driven cell proliferation as a neoplasm-specific dependency [44–47].

While FACT did not initially seem essential for cell proliferation outside of the context of cancer, more recent work has demonstrated heightened FACT expression and novel requirement in undifferentiated (stem) cells [43–48]. Stem cell chromatin is highly regulated by well-characterized features, including an accessible chromatin landscape relative to other cell types and bivalent chromatin, which is epigenetically decorated with both active (e.g., H3K4me3) and repressive (e.g., H3K27me3) modifications [49–57]. Embryonic stem (ES) cells specifically regulate their chromatin to prevent differentiation from occurring until appropriate, thereby preserving their pluripotent state. Pluripotency, or the capacity to mature into most cell types in an adult organism, is maintained by a suite of master regulators that work to repress differentiation-associated genes and maintain expression of genes that promote this pluripotent state. Factors required for this state in embryonic stem cells include the well-studied transcription factors OCT4, SOX2, KLF4, MYC, and NANOG, which are often referred to as master regulators of pluripotency [57–65]. The main functions of these factors are to maintain pluripotency and prevent improper differentiation through regulation of gene expression; however, a majority of their chromatin interactions occur at gene-distal genomic regions such as enhancers [66]. FACT has been shown to interact with several pluripotency- and development-associated factors, including OCT4 [60, 61], WNT [67], and NOTCH [60, 61, 68]. In addition, FACT has been functionally implicated in maintaining stem cells in their undifferentiated state [44, 45, 47]. *Ssrp1* shRNA knockdown led to faster differentiation into neuronal precursor cells relative to control, along with increased expression of genes involved in neural development and embryogenesis [47]. In both *C. elegans* and mammalian fibroblasts, FACT was shown to impede transition between pluripotent and differentiated states; in *C. elegans*, FACT was identified as a barrier to cellular reprogramming of germ cells into neuronal precursors, while in human fibroblasts and murine embryonic fibroblasts, FACT inhibition prevented reprogramming to induced pluripotent stem cells [44, 45]. These experiments have confirmed a dependency for FACT in pluripotent cells that is not found in differentiated fibroblasts [44, 45]. While these data establish a role for FACT in pluripotent cells, the mechanism through which FACT acts within undifferentiated cells to maintain their state is currently unclear.

To probe the function of FACT in ES cells, we utilized rapid depletion through auxin-inducible degron (AID) tagging of SPT16, followed by transcriptomic and chromatin studies. We find that SPT16 binds to both promoter and gene-distal cis-regulatory elements and that nearly 60% of FACT binding sites overlap with those of master regulators of pluripotency (OCT4, SOX2, and NANOG). Acute SPT16 depletion resulted in reduced transcription at highly transcribed genes, but also caused increased transcription of some genes. Furthermore, we identify extensive regulation of non-coding transcription by the FACT complex at cis-regulatory elements. SPT16 binding is highly enriched at putative enhancers, and acute SPT16 depletion was found to alter transcription of putative enhancer RNAs (eRNAs), including eRNAs transcribed from enhancers of *Pou5f1*,* Sox2,* and *Nanog.* Furthermore, we show that FACT is required to maintain accessible chromatin at binding sites shared by FACT and master regulators of pluripotency. We propose a model whereby FACT functions at regulatory elements to maintain open chromatin structure, allowing binding of pluripotency master regulators and expression of genes required for pluripotency.

## Results

### Inducible depletion of the FACT complex triggers a reduction in pluripotency

**Fig. 1:**
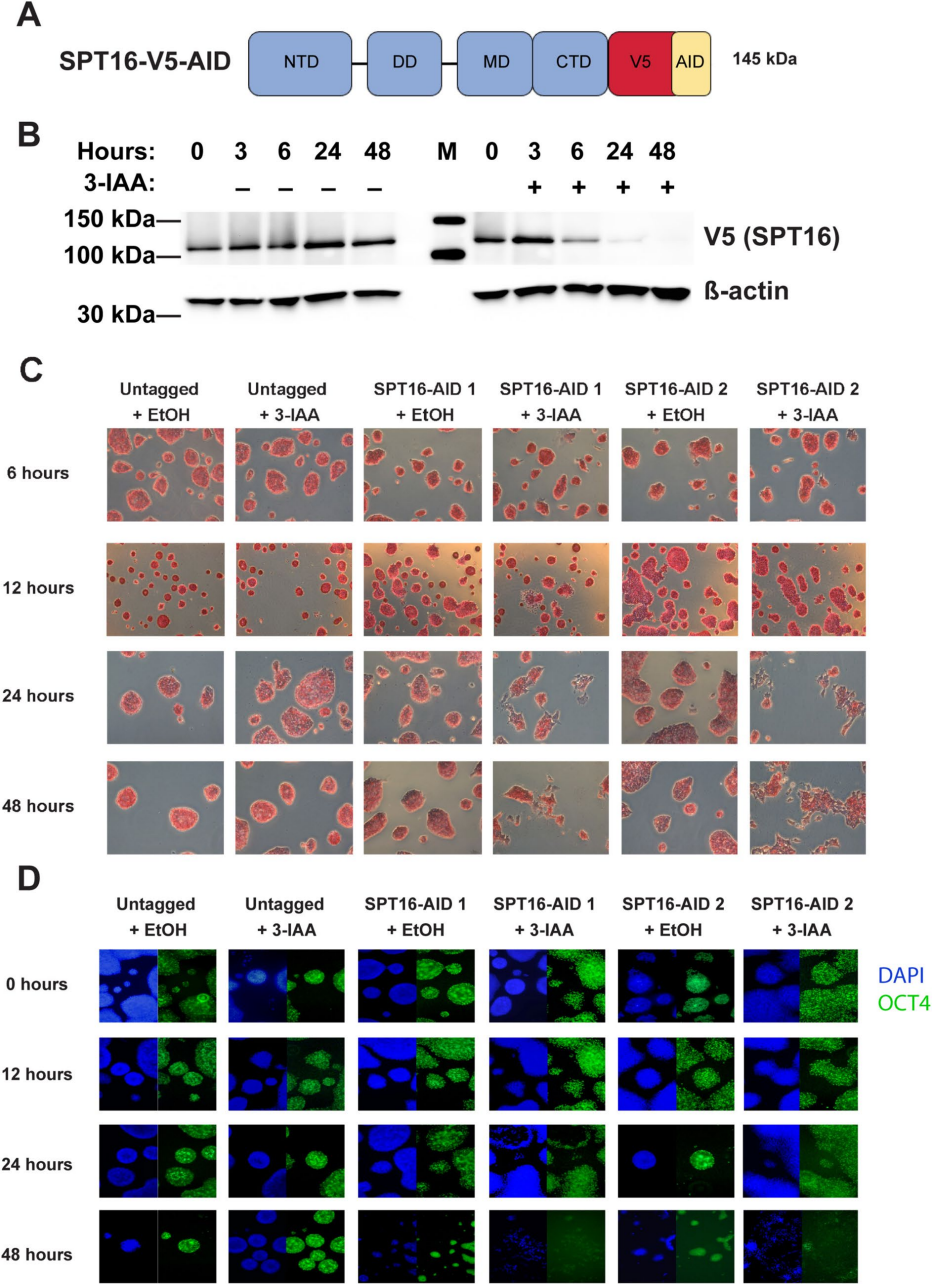
Inducible depletion of SPT16 triggers reduced pluripotency in ES cells. **A** Schematic of auxin-inducible degron (AID) and V5-tagged SPT16 protein. NTD = N-terminal domain, DD = dimerization domain, MD = middle domain, CTD = C-terminal domain, AID = minimal auxin-inducible degron tag, V5 = 3xV5 epitope tag. **B** Western blot showing depletion of SPT16 after 0, 3, 6, 24, and 48 h treatments with IAA (+) or vehicle control (EtOH, −). 40 µg total protein loaded per lane. Top: V5 antibody (for tagged SPT16) and bottom: β-actin antibody. Representative blot shown from SPT16-V5-AID clone 1; additional blots can be found in Additional File 1: Fig. S1. “M” denotes a molecular weight marker lane. **C** Timecourse of IAA or EtOH treatment for 6, 12, 24, or 48 h to deplete SPT16 showing morphological changes following alkaline phosphatase staining. Images are representative of plate-wide morphological changes. Alkaline phosphatase staining is quantified in Additional File 1: Fig. S1E. D. Timecourse of IAA or EtOH treatment for 12, 24, or 48 h to deplete SPT16, followed by immunocytochemistry showing a progressive reduction in the expression of OCT4. Images are representative of plate-wide immunofluorescence changes. Left panels are DAPI-stained (blue), while right panels show OCT4 immunofluorescence (green). OCT4 immunofluorescence is quantified in Additional File 1: Fig. S1F

To examine the role of FACT in ES cells, we acutely depleted the FACT subunit SPT16 via the auxin-inducible degron (AID) system in murine embryonic stem (ES) cells grown in naïve conditions (serum/LIF + 2i). Briefly, we used CRISPR/Cas9-directed homologous recombination to insert a mini-AID and 3XV5 tag at the 3′ end of endogenous *Supt16h,* the gene encoding SPT16, in ES cells that have osTIR1 already integrated within the genome (see “Methods,” Fig. 1A). Relative to vehicle treatment (EtOH), SPT16 protein levels were reduced to minimal levels by proteasomal degradation following 24-h treatment with the auxin 3-indole acetic acid (3-IAA or IAA), and partially reduced after 6 h (Fig. 1B, Additional File 1: Fig. S1A). We note that, in line with previous reports, SSRP1 protein levels are reduced upon depletion of SPT16 (Additional File 1: Fig. S1B, S1C) [69].

Consistent with a role for FACT in stem cell identity and viability, within 24 h of IAA addition, ES cell colonies show phenotypic changes indicative of cellular differentiation, including reduced alkaline phosphatase activity, a reduction in OCT4 protein expression, and morphological changes relative to vehicle treated or untagged cells (osTIR1 expressing cells in which SPT16 lacks an AID tag; Fig. 1C,D, Additional File 1: Fig. S1D-F) [70]. These phenotypic changes were most apparent between 24- and 48-h IAA treatment, while at earlier timepoints there are few phenotypic changes, likely due to incomplete SPT16 depletion at these earlier timepoints. While it has been suggested that stem cells require FACT as a result of cellular stress induced by trypsinization [44], we note that cells were left undisturbed for 48 h prior to protein depletion, implying that trypsinization is unrelated to FACT-depleted differentiation or viability.

### SPT16 occupancy is enriched at pluripotency factor binding sites

**Fig. 2:**
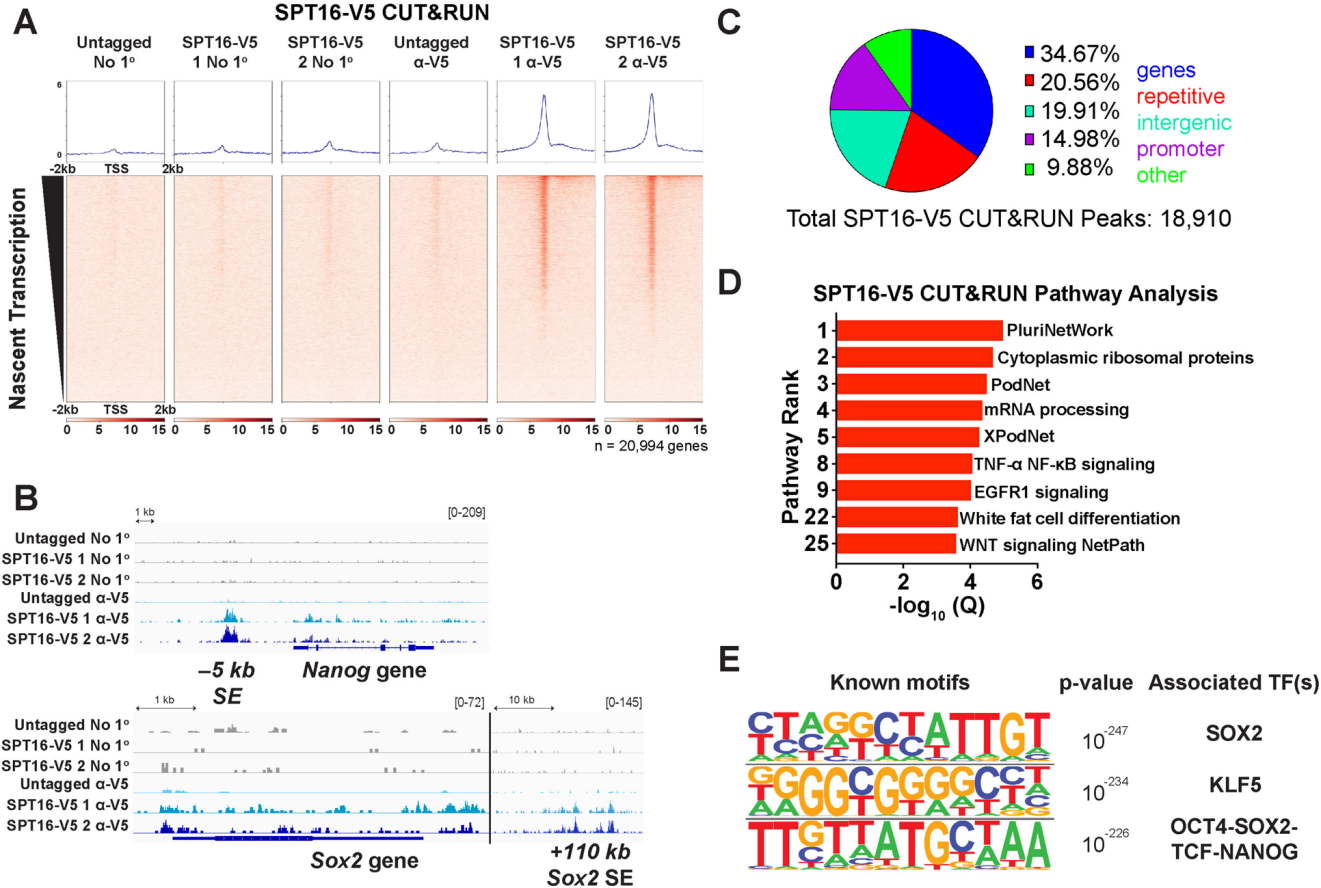
SPT16 is enriched at sites occupied by master regulators of pluripotency. **A** SPT16-V5 CUT&RUN data visualized over transcription start sites and sorted by nascent transcription in control samples (TT-seq data; see Fig. 3). Averaged replicates are shown as heatmaps ± 2 kb from the TSS (*n* = 3 for untagged, *n* = 2 for each V5-tagged clone). No 1º refers to negative control experiments where no primary antibody is added, but pA/G-MNase is still added to assess background cutting. **B** IGV genome browser track for CUT&RUN data at the *Nanog* (top) and *Sox2* (bottom) loci. Averaged replicates are shown as a single track (*n* = 3 for untagged and *n* = 2 for each V5-tagged clone). **C** Proportion of peaks called from V5-enriched CUT&RUN corresponding to gene bodies (blue), repetitive regions (red), intergenic regions (teal), promoters (purple, defined as 1 kb upstream of annotated TSSs), and other regions (green). **D** Pathway analysis of SPT16-V5 CUT&RUN peaks overlapping mRNA loci (genic and promoter regions). Q-values were calculated from *p*-values by the Benjamini–Hochberg procedure. **E** The three most significantly enriched sequence motifs of all SPT16-V5 CUT&RUN peaks (*n* = 4) identified using HOMER [74]

To determine where FACT is acting throughout the genome, we performed chromatin profiling using CUT&RUN [71] on the endogenously tagged SPT16-V5 protein. Attempts to profile SPT16 or SSRP1 with commercial antibodies directly targeting the proteins were unsuccessful in our hands. SPT16-V5 binding at genes correlated with transcription (Fig. 2A), as expected based on its known functions in transcription elongation [38, 47, 72]. Interestingly, not only was SPT16 enriched at pluripotency-regulating genes, such as *Nanog* and *Sox2*, SPT16 binding was also elevated at distal enhancer elements (Fig. 2B). We called peaks using SEACR [73, 74] and examined localization to genomic features (Fig. 2C). We find that FACT localizes mostly to transcribed regions (~ 50% of peaks are promoter or genic) with numerous intergenic peaks. We subjected genic SPT16 peaks to Gene Ontology (GO) term analysis, identifying numerous pluripotency- and development-associated pathways (Fig. 2D). To assess the association between FACT and pluripotency orthogonally, we performed sequence motif analysis of all CUT&RUN peaks using HOMER (Fig. 2E) [74]. The top three most enriched sequence motifs were those recognized by the transcription factors SOX2, KLF5, and OCT4-SOX2-TCF-NANOG, all of which regulate cellular pluripotency or differentiation [57, 61–65, 75]. Together, these results suggest that FACT may maintain pluripotency of ES cells in part through co-binding of target genes with the master regulators of pluripotency.

Because the specific culture conditions in which murine ES cells are grown can alter cellular dynamics [76], we performed SPT16-V5 CUT&RUN on cells grown in both 2i + LIF (shown in Figs. 2 and S2A, left) and LIF alone (Additional File 1: Fig. S2A, right). While intensity of binding was reduced in cells grown in LIF alone, overall binding trends were similar between experiments. To further inform our subsequent experiments, we also compared public ChIP-seq data for pluripotency factors OCT4, SOX2, and NANOG grown in 2i + LIF or LIF only conditions from GSE56312 [76] and GSE174774 [77] (Additional File 1: Fig. S2B [76–78]). We visualized these data at peaks called from SPT16-V5 CUT&RUN (2i + LIF) and found strong and consistent binding of all three examined pluripotency factors (Additional File 1: Fig. S2B, left, center). We also visualized these data over LIF-alone OCT4 ChIP-seq peaks from GSE11724 [78] and found few distinctions between culture conditions (Additional File 1: Fig. S2B, right). As spontaneous differentiation occurs less often in ground-state ES cells, experiments performed in 2i + LIF require a higher threshold of effect to trigger cell differentiation. Therefore, we performed all subsequent experiments using cells cultured in 2i + LIF.

### FACT regulates expression of the master regulators of pluripotency

**Fig. 3:**
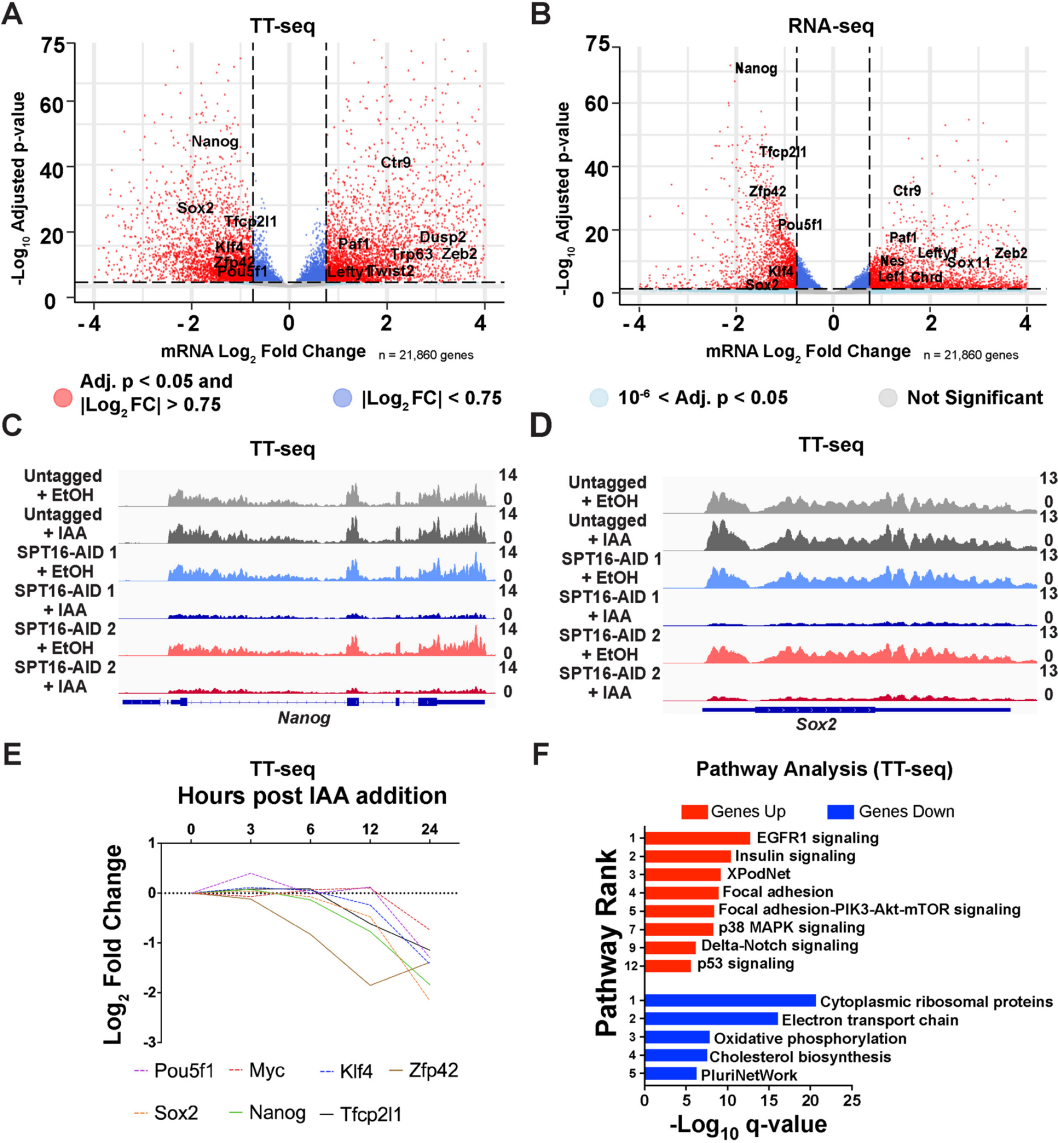
Depletion of FACT disrupts transcription of pluripotency factors. **A** Volcano plot of differential mRNA transcription after 24-h treatment (TT-seq, analyzed with DESeq2). Red points indicate significant changes (adj. *p* < 0.05, |log_2_ fold change|> 0.75). Light blue points are significant changes by adjusted *p*-value but below the fold change cutoff, while dark blue points are significant changes by log_2_ fold change but below the adjusted *p*-value cutoff (for plotting, adj. *p* < 10^−6^). **B** Volcano plot of differential mRNA abundance after 24-h treatment (traditional RNA-seq, analyzed with DESeq2). Points are colored as in panel **A**. **C** IGV genome browser tracks showing nascent transcription (TT-seq) from the *Nanog* gene following 24-h IAA treatment to deplete SPT16. Averaged replicates are shown as a single track, oriented to the genic strand (*n* = 3). **D** As in **B** but visualized over the *Sox2* gene locus. **E** DESeq2 results: mRNA transcription of seven pluripotency factors across depletion timecourse. Significance of altered pluripotency factor transcription was analyzed by Friedman tests and corrected for multiple comparisons via Dunn’s test (*p* = 0.0027). **F** Pathway analysis of differentially expressed genes following 24-h IAA treatment to deplete SPT16. *Y*-axis indicates enrichment ranking. *Q*-values were calculated from *p*-values by the Benjamini–Hochberg procedure

We next examined the requirement for FACT in gene transcription in ES cells. We depleted SPT16 and performed nascent RNA sequencing (TT-seq) as a direct readout of transcription that is relatively free from the confounding effects of RNA processing and mRNA stability [79]. To assess the effects of SPT16 depletion on transcription over time, we performed a timecourse of 0-, 3-, 6-, 12-, and 24-h IAA treatment. We observed few differentially transcribed genes prior to strong SPT16 depletion (0–6 h; Table 1, Additional File 1: Fig. S3A). RT-qPCR using total RNA isolated at 3- or 6-h IAA treatment confirmed that *Pou5f1*,* Sox2*, or *Nanog* transcript levels had not changed, consistent with the morphological studies and nascent transcriptomics (Additional File 1: Fig. S3B-D). Beginning with 12-h IAA treatment, we observed a reduction in pluripotency factor expression and increased expression of differentiation markers (Fig. 3A–E, S3E-H, Additional File 2: Table S1). These trends were validated by comparison with traditional steady-state RNA-seq (Fig. 3B, Table 1, Additional File 2: Table S1), which identified similar changes to pluripotency and differentiation factor expression. As pluripotency factor expression does not decline prior to strong depletion of SPT16, we infer that FACT activity is required to maintain expression of these important transcription factors; in support of this hypothesis, the rate of differentiation is accelerated in the absence of SPT16 (phenotypes arising within 24 h [Fig. 1C]), as opposed to standard mES cell differentiation methods that show signs of differentiation only after 3–5 days of LIF removal [80]. Cells depleted of SPT16 for only 12 h were unable to recover to a pluripotent state upon IAA washout (Table 1, Additional File 2: Table S1), potentially placing FACT in a gatekeeping role between pluripotent and differentiated cells, in line with previous reports [44, 45, 47, 81].

**Table 1:** SPT16 depletion alters mRNA transcription and abundance, with most prominent effects after 12 + h of depletion

Hours depleted	0	3	6	12	12 + 24-h washout	24	24 [RNA-seq]
**mRNAs up**	3	58	214	1366	1815	5398	3977
**mRNAs down**	53	5	174	1932	1427	5000	3781

Control samples and SPT16-depleted samples were separately pooled between cell lines for downstream analyses. Only transcripts with an adjusted *p*-value of < 0.05 are displayed (analyzed with DESeq2)

Modest depletion of SPT16 (≤ 6-h IAA treatment) is accompanied by more genes with increased transcription than genes with decreased transcription (Additional File 1: Fig. S3A, S4, Table 1). However, we identified more genes with decreased transcription than increased transcription at the two subsequent time points (Fig. 3A, S3A, S4, Table 1). Of the genes encoding master pluripotency factors, only *Nanog* was significantly reduced at 12-h IAA treatment (Fig. 3E, Additional File 2: Table S1), while *Pou5f1*, *Sox2, Klf4, Myc*, and *Nanog* were significantly reduced by 24 h (Fig. 3A–E, Additional File 1: Fig. S3E, S3I, Additional File 2: Table S1). We observe enrichment of SPT16-V5 binding over FACT-regulated genes, with little enrichment over genes with unchanged expression upon SPT16 depletion (Additional File 1: Fig. S5A-F, Additional File 2: Table S1) [82–84].

To identify cellular processes critically regulated by FACT, we subjected differentially transcribed genes at 24-h IAA treatment to pathway analysis and identified enrichment for the pluripotency network among genes with reduced transcription, while numerous signaling pathways were enriched among genes with increased transcription (Fig. 3F). Differentially transcribed genes were compared to a background list of all transcribed genes (DESeq2 baseMean of ≥ 1) to confirm that pathways were enriched in differential transcripts, rather than the result of cell-type-specific expression. We infer a dependency of the master regulators on FACT where upon FACT depletion, ES cells are forced to differentiate more rapidly as OCT4, SOX2, and NANOG (hereafter referred to collectively as “OSN”) expression is not maintained. Together, these data show a progressive reduction of pluripotency factor expression over the timecourse of FACT depletion.

### FACT binds to gene-distal putative enhancers and not putative silencers

**Fig. 4:**
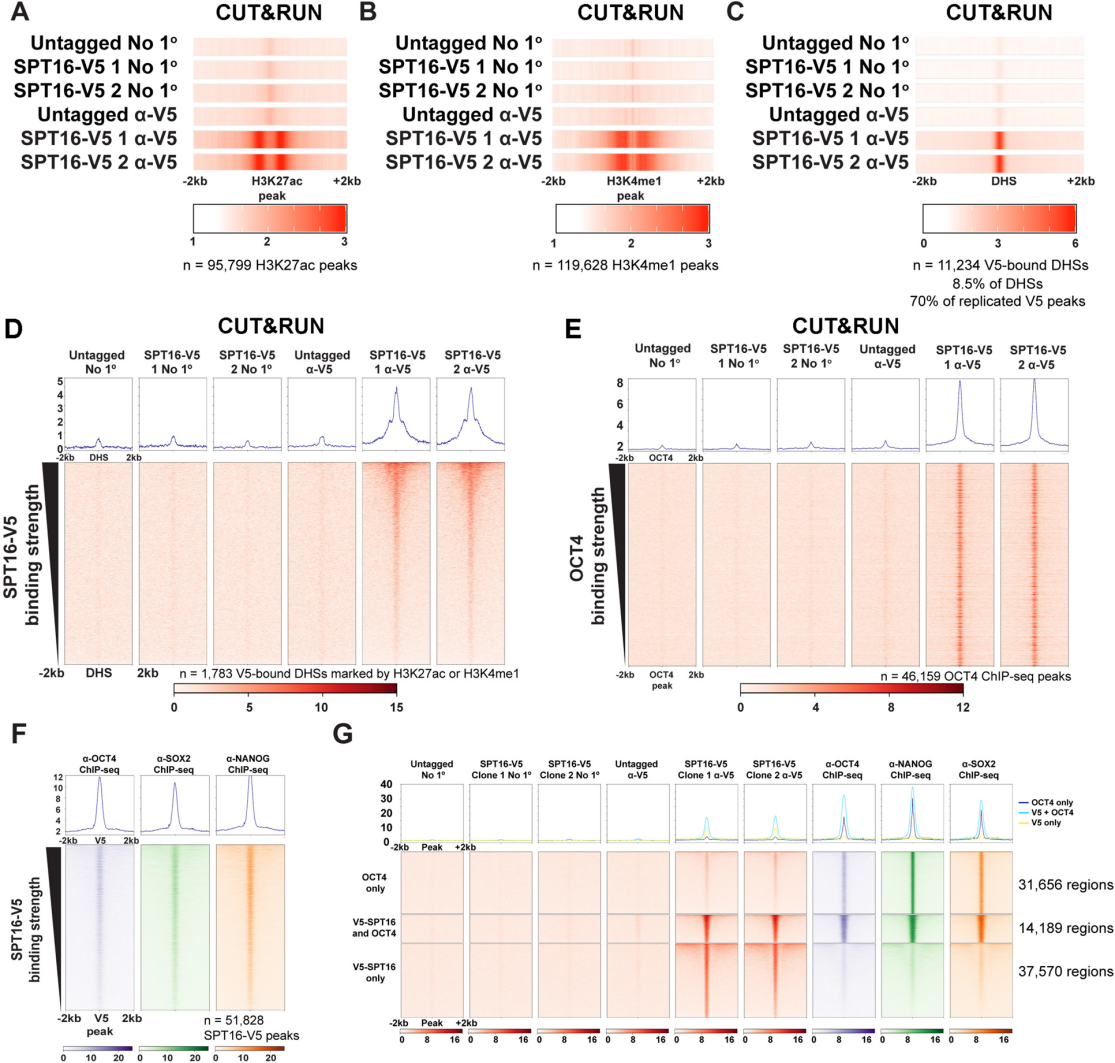
FACT and pluripotency factors colocalize at putative enhancers genome-wide. **A**–**C** SPT16-V5 CUT&RUN data visualized as one-dimensional heatmaps [47]. Each row represents the average of technical replicates, while biological replicates are displayed separately (*n* = 3 for untagged and *n* = 2 for each V5-tagged clone). Visualized at **A** H3K27ac ChIP-seq peaks ± 2 kb (GSE32218), **B** H3K4me1 ChIP-seq peaks ± 2 kb (GSE31039), **C** TSS-distal DNaseI hypersensitive sites ± 2 kb (GSM1014154) [83–85]. **D** SPT16-V5 CUT&RUN data visualized at SPT16-V5-bound putative enhancers, defined as DHSs (GSM1014154) overlapping H3K4me1 or H3K27ac ChIP-seq peaks (GSE32218 and GSE31039) ± 2 kb [83–85]. **E** SPT16-V5 CUT&RUN binding over TSS-distal OCT4 ChIP-seq peaks (ChIP-seq from GSE11724 [78]). Merged replicates are shown as heatmaps ± 2 kb from the center of the OCT4 ChIP-seq peak (*n* = 3 for untagged, *n* = 2 for each V5-tagged clone). **F** OCT4, SOX2, or NANOG enrichment over SPT16-V5 CUT&RUN peaks. Averaged replicates shown (*n* = 1 for OCT4, *n* = 2 for SOX2 and NANOG; ChIP-seq from GSE11724) [78]. Significance of overlap between pluripotency factor binding and SPT16-V5 binding was assessed via two-tailed Fisher’s exact tests (*p* = 0 for all remodelers, ratio = 61.597, 94.475, and 93.779 for OCT4, SOX2, and NANOG overlap with SPT16-V5, respectively). **G** Heatmaps displaying overlap between OCT4 ChIP-seq and SPT16-V5 CUT&RUN peaks. Clusters were assigned by direct overlap between peak datasets and are individually sorted by strength of SPT16-V5 binding (ChIP-seq from GSE11724) [78]. Merged replicates are shown as heatmaps ± 2 kb from the center of the peak (*n* = 3 for untagged, *n* = 2 for each V5-tagged clone, *n* = 1 for OCT4 ChIP-seq, *n* = 2 for other ChIP-seq experiments; ChIP-seq from GSE11724) [78]

In addition to gene-proximal (promoter and gene body) regions, we found that SPT16 also binds to thousands of gene-distal (intergenic) genomic regions (Fig. 2C), including known enhancers of *Nanog* and *Sox2* (Fig. 2B). Therefore, we assessed SPT16 binding at sites enriched for a number of features prominent at active enhancers, including H3K27ac (Fig. 4A), H3K4me1 (Fig. 4B), and TSS-distal DHSs (Fig. 4C). FACT is enriched at each of these sites (Fig. 4A-C), as well as over TSS-distal DHSs also decorated with H3K27ac and/or H3K4me1 (Fig. 4D). Although FACT binds numerous DHSs, we note that SPT16 binding is not enriched at putative silencers, defined by the presence of a TSS-distal DHS and a H3K27me3 ChIP-seq peak (Additional File 1: Fig. S5G-I). Together, these data indicate FACT does not bind indiscriminately to all accessible regions, but preferentially binds at promoters and putative enhancers.

### SPT16 co-occupies TSS-distal regions bound by OCT4, SOX2, and NANOG

Given that SPT16 occupies putative enhancers and is essential for maintenance of pluripotency, we hypothesized that SPT16 may colocalize with transcription factors required for pluripotency. To test this possibility, we quantified overlap between OSN ChIP-seq peaks [78] and SPT16-V5 CUT&RUN peaks and found that ~60% of SPT16 peaks overlap with peaks corresponding to OCT4, SOX2, or NANOG (Table 2). Supporting this peak-based analysis, SPT16-V5 CUT&RUN shows strong enrichment over TSS-distal OCT4 ChIP-seq peaks (Fig. 4E). Orthogonally, we analyzed published OCT4, SOX2, and NANOG ChIP-seq data and visualized enrichment for these factors over SPT16-V5 CUT&RUN peaks (Fig. 4F–G) [78]. All three pluripotency factors display enriched binding over SPT16-V5 binding sites, supporting colocalization of FACT and pluripotency factors.

**Table 2:** Overlap between SPT16-V5 CUT&RUN peaks and OCT4, SOX2, and NANOG ChIP-seq peaks

Peak overlap category	OCT4 peaks	Sox2 Peaks	Nanog Peaks	Any of OSN
**V5 Peaks**	8544	5948	5307	9682
**OSN Peaks**	45,476	19,211	16,817	52,899
**% of OSN peaks bound by FACT**	18.33%	30.72%	31.40%	17.91%
**% of V5 peaks bound by OSN**	52.41%	36.85%	32.94%	59.63%
**V5-bound promoters**	4261	2719	2327	4452
**OSN-bound promoters**	6550	1542	666	6948
**V5- and OSN-bound promoters**	2040	801	343	2202
**OSN-bound TSS-distal peaks**	38,926	17,669	16,151	45,938
**V5-bound TSS-distal OSN peaks**	6504	5147	4964	7480

To be called as overlapping, peaks must have directly shared at least 1 bp and passed peak-calling thresholds (see “Methods”)

### SPT16 depletion alters non-coding transcription at gene-distal regulatory sites

**Fig. 5:**
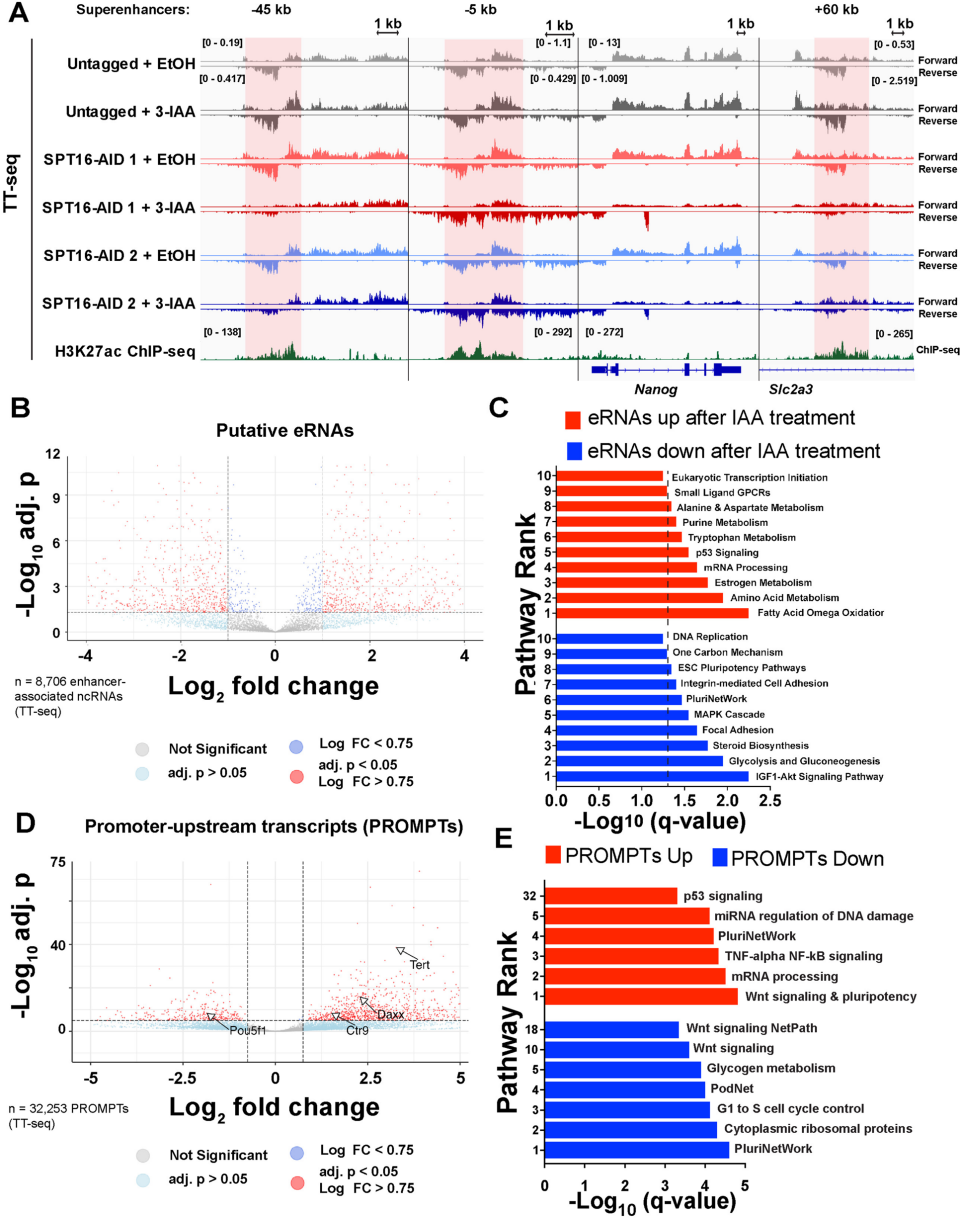
TT-seq identifies FACT-dependent regulation of non-coding RNAs. **A** IGV genome browser tracks showing nascent transcription (TT-seq) over the *Nanog* gene and three *Nanog* superenhancers following 24-h IAA treatment to deplete SPT16, along with published H3K27ac ChIP-seq data. Three individually scaled windows are shown to highlight eRNA transcription from the superenhancers (shaded red) and *Nanog* gene (shaded blue). Merged replicates are shown as a single track (TT-seq: *n* = 3, H3K27ac ChIP-seq: *n* = 1; ChIP-seq from GSE32218) [83–85]. **B** Volcano plot of differentially transcribed putative eRNAs (analyzed with DESeq2). Putative eRNAs were called by transcription from a TSS-distal DHS marked by either H3K4me1 or H3K27ac. Red points are significantly changed ncRNAs (adj. *p* < 0.05, log_2_ fold change > 0.75). Dark blue points are significantly changed by adjusted *p*-value but below the fold change cutoff, while light blue points are significantly changed by log_2_ fold change but below the adjusted *p*-value cutoff. Labeled arrows denote closest genes to the indicated ncRNA. **C** Pathway analysis of nearest genes to differentially expressed putative eRNAs following 24-h IAA treatment to deplete SPT16. *Y*-axis indicates pathway enrichment ranking. *Q*-value was calculated from *p*-values by the Benjamini–Hochberg procedure. **D** As in **B**, but for differentially expressed PROMPTs. **E** As in **C**, but for the genes nearest differentially expressed PROMPTs

Active enhancers are often sites of non-protein-coding transcription, producing enhancer RNAs (eRNAs), which are thought to play roles in activation of coding gene expression [86–90]. We therefore sought to determine whether FACT localization to TSS-distal OCT4-, SOX2-, and/or NANOG-bound putative enhancers may regulate non-coding transcription known to arise from these regions. Using our timecourse of SPT16 depletion followed by TT-seq, we identified FACT-dependent transcription of eRNAs from known superenhancers of the *Pou5f1*,* Sox2*, and *Nanog* genes (Fig. 5A, Additional File 1: Fig. S6A-B). To examine eRNA regulation more globally, we examined transcription changes at all putative enhancers. Out of 70,586 TSS-distal regulatory regions (defined as TSS-distal DHSs), 57,954 were sites of nascent transcription detected in our control TT-seq datasets (Table 3, Additional File 1: Fig. S3A, Additional File 1: Fig. S4). We identified 14,532 ncRNAs (26%) regulated by FACT, with more ncRNAs upregulated (15%, 8,743) than downregulated (11%, 5789) following 24-h IAA treatment (Table 3, Additional File 1: Fig. S3A, Additional File 1: Fig. S4). Taking only the ncRNAs transcribed from regions marked by both a TSS-distal DHS and either H3K4me1 or H3K27ac as putative eRNAs, we identified 11,964 transcripts, with 18% of these putative eRNAs upregulated (2701) and 16% downregulated (2439) upon 24-h IAA treatment (Table 3, Fig. 5B, Additional File 1: Fig. S4). Assuming that gene-distal ncRNAs regulate expression of the nearest gene, we performed pathway analysis on putative mRNA targets (Fig. 5C). Again, we used a background list of all transcribed genes (DESeq2 baseMean ≥ 1) to filter preferential enrichment of genes expressed in ES cells. As with differentially expressed coding genes, among the most significantly enriched categories for putative targets of upregulated eRNAs were mechanisms associated with pluripotency, while putative targets of downregulated ncRNAs were also enriched for pluripotency genes, along with signaling pathways.

**Table 3:** Significantly altered coding and non-coding RNAs at 0, 3, 6, 12, and 24 h of SPT16 depletion

Hours depleted	0	3	6	12	24
**mRNAs up**	3	58	214	1366	5398
**mRNAs down**	53	5	174	1932	5000
**DHS-associated ncRNAs up**	0	4	38	203	8743
**DHS-associated ncRNAs down**	0	0	34	323	5789
**Putative eRNAs up**	0	78	137	154	2701
**Putative eRNAs down**	0	0	21	122	2439
**PROMPTs up**	0	4	4	95	2984
**PROMPTs down**	0	0	0	59	1831

Control samples and SPT16-depleted samples were separately pooled between cell lines for downstream analyses. Only transcripts with an adjusted *p*-value of < 0.05 are displayed (analyzed with DESeq2). Data were analyzed for significance via a two-way ANOVA and corrected for multiple comparisons using Dunnett’s test. Significant differences between transcript class were not identified (*p* = 0.3579, 5.077% of total variation), while each class of transcripts was significantly altered over depletion time (*p* < 0.0001, 77.58% of total variation)

We next sought to determine whether FACT regulates another class of cis-regulatory element ncRNAs, those produced at promoters divergent from mRNAs, termed promoter upstream transcripts (PROMPTs; also referred to as upstream antisense non-coding RNAs or uaRNAs). PROMPTs were identified by genomic location (within 1 kb of an annotated TSS and transcribed divergently to the mRNA). We identified 4815 PROMPTs out of 23,256 expressed putative PROMPTs with significantly altered transcription after 24-h IAA treatment (adj. *p* < 0.05; Fig. 5D). PROMPTs were assigned to nearest genes (detailed in “Methods”) and those with altered expression were subjected to pathway analysis. Indeed, we identified enrichment of pluripotency- and differentiation-associated pathways (Fig. 5E). Together these data show substantial regulation of non-coding RNA transcription by FACT, with pronounced effects on ncRNAs associated with pluripotency factors—particularly their associated eRNAs (Fig. 5A, Additional File 1: Fig. S6).

### SPT16 depletion leads to increased nucleosome occupancy over FACT-bound locations

**Fig. 6:**
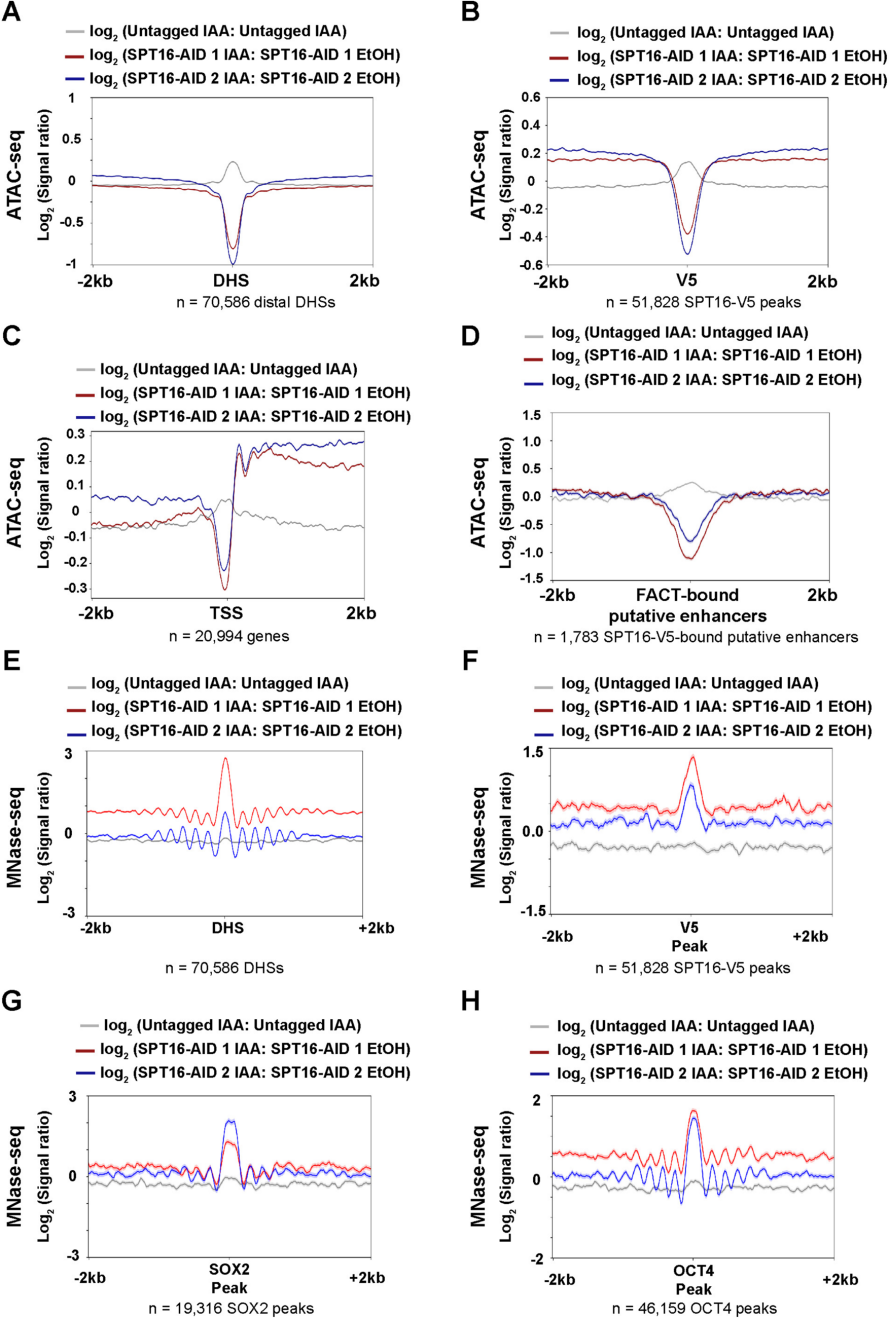
FACT depletion has distinct effects on chromatin accessibility and nucleosome occupancy at SPT16-V5 binding sites and gene regulatory regions. **A** Differential chromatin accessibility visualized over TSS-distal DHSs, ± 2 kb, after 24-h IAA treatment. Higher signal indicates more accessible chromatin in IAA-treated samples than in EtOH-treated samples at the indicated timepoint, with the exception of untagged samples (log_2_ IAA:IAA ratio) (*n* = 1 per cell line). Significance of altered chromatin accessibility at gene-distal DHSs (**A**) was analyzed by Friedman tests and corrected for multiple comparisons via Dunn’s test (*p* < 0.0001 overall, adj. *p* < 0.0001 for each individual comparison). **B** As in **A** but visualized over SPT16-V5 binding sites identified in Fig. 2. Significance of altered chromatin accessibility at SPT16-V5 binding sites was analyzed as in panel **A** (*p* < 0.0001 overall, adj. *p* < 0.0001 for each individual comparison). **C** As in **A** but visualized over RefSeq mRNA TSSs, ± 2 kb, after 24-h IAA treatment. Significance of altered chromatin accessibility was analyzed as in panel **A** (*p* < 0.0001 overall, adj. *p* < 0.0001 for both individual comparisons to Untagged. **D** Metaplot depicting change in chromatin accessibility at 24-h treatment over putative enhancer regions as defined in Fig. 5. Standard error is shaded in either direction. Significance of altered chromatin accessibility was analyzed as in panel **A** (*p* < 0.0001 overall, adj. *p* < 0.05 for each individual comparison except Untagged [adj. *p* = 0.4046]). **E**–**H** Differential nucleosome occupancy following 24-h IAA treatment (MNase-seq, *n* = 3 for untagged samples, *n* = 2 for each tagged cell line). Visualized over **E** TSS-distal DNaseI hypersensitive sites (DHSs) ± 2 kb (DNase-seq from GSM1014154) [83–85]. Significance of altered chromatin accessibility was analyzed as in panel **A** (*p* < 0.0001 overall, adj. *p* < 0.001 for each individual comparison). **F** Peaks called from SPT16-V5 CUT&RUN ± 2 kb. Significance of altered chromatin accessibility was analyzed as in panel **A** (*p* < 0.0001 overall, adj. *p* < 0.0001 for each individual comparison). **G** SOX2 ChIP-seq binding sites, ± 2 kb (ChIP-seq from GSE11724) [78]. Significance of altered chromatin accessibility was analyzed as in panel **A** (*p* < 0.0001 overall, adj. *p* < 0.0001 for each individual comparison). **H** OCT4 ChIP-seq binding sites, ± 2 kb (ChIP-seq from GSE11724) [78]. Significance of altered chromatin accessibility was analyzed as in panel **A** (*p* < 0.0001 overall, adj. *p* < 0.0001 for each individual comparison)

As FACT is a histone chaperone that can remove histone H2A/H2B dimers, we hypothesized that FACT may regulate chromatin accessibility through nucleosome clearance which ultimately drives transcription. To identify changes in chromatin accessibility upon FACT depletion, we performed ATAC-seq across a 3-, 6-, 12-, and 24-h timecourse of IAA treatment. Consistent with the localization trends described in Fig. 4, FACT depletion leads to decreased accessibility directly over TSS-distal DHSs, SPT16-V5-bound sites, and promoters after 12- and 24-h IAA treatment, with trends strengthening over time (Fig. 6A–C, Additional File 1: Fig. S7A-C, S8). Furthermore, we observe a decrease in chromatin accessibility directly over SPT16-bound TSS-distal DHSs, indicating that FACT is necessary for maintenance of accessible chromatin at putative enhancers (Fig. 6D). These effects are not recovered by 24-h washout of IAA from 12-h-depleted cells (Table 1, Additional File 2: Table S1, Additional File 1: Fig. S9), suggesting that short-term SPT16 depletion alone can induce terminal cell differentiation, even with rescue of SPT16 expression. Together, these data suggest a mechanism of SPT16-dependent nucleosome clearance, wherein FACT assists in maintaining accessible chromatin at gene-distal regulatory elements in ES cells.

For a more precise understanding of changes to nucleosome occupancy and positioning upon SPT16 depletion, we performed micrococcal nuclease digestion followed by deep sequencing (MNase-seq) following 24-h IAA treatment. In agreement with the ATAC-seq results (Fig. 6A–C), we observed increased nucleosome occupancy directly over promoter and gene-distal regions following SPT16 depletion (Fig. 6E, Additional File 1: Fig. S7D-E, Additional File 1: S10) [78, 83, 84]. An increase in nucleosome occupancy following SPT16 depletion is also observed over SPT16-V5 peaks (Fig. 6F, Additional File 1: Fig. S10A), and at OSN binding sites (Fig. 6G–H, Additional File 1: Fig. S7F, Additional File 1: Fig. S10C-E). Together with the ATAC-seq results, these data demonstrate SPT16 is necessary to maintain open, nucleosome-free chromatin at promoters and gene-distal regulatory sites, including those bound by OSN.

## Discussion

**Fig. 7:**
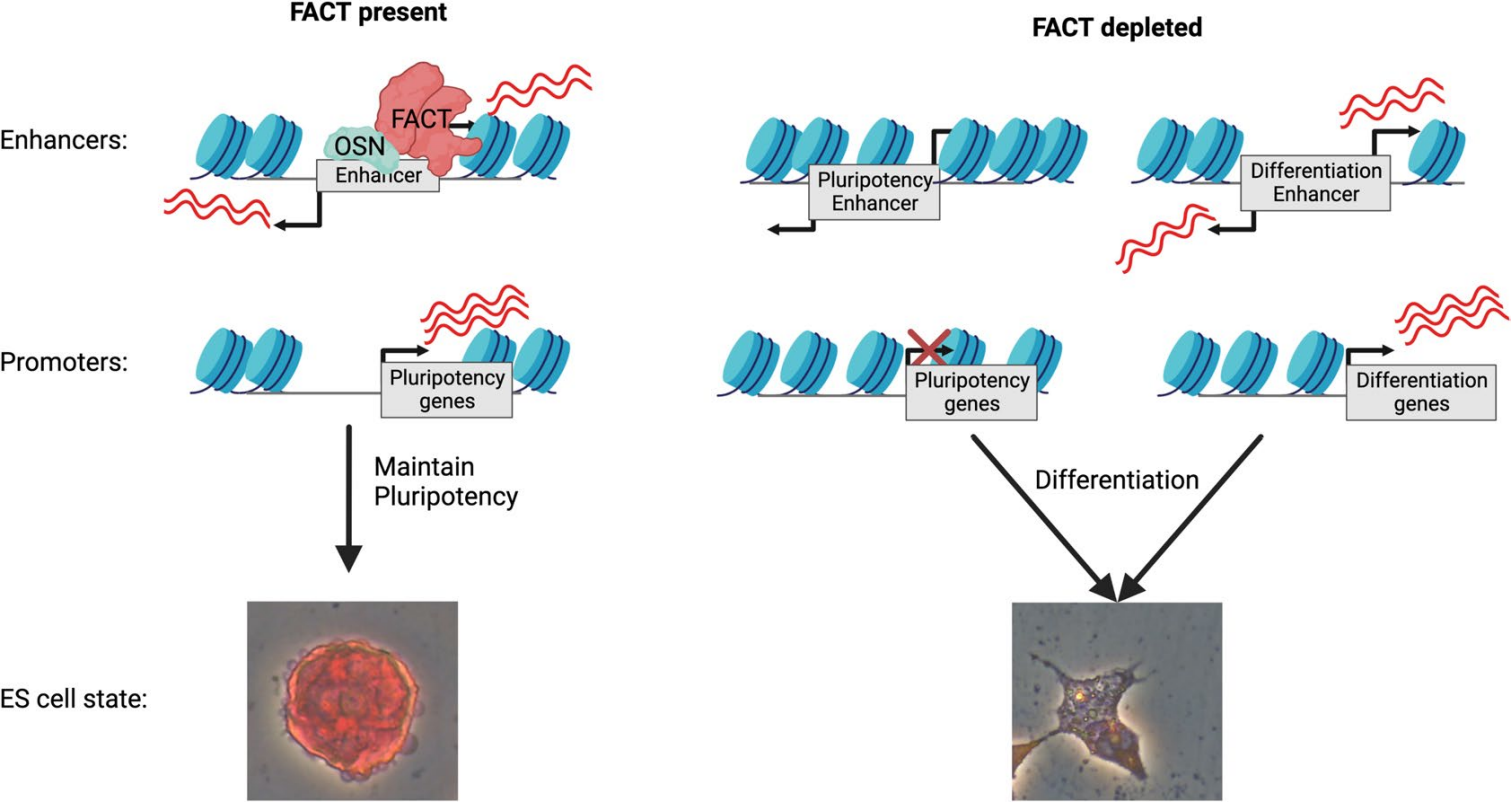
Working model: FACT maintains ES cell pluripotency through regulation of pluripotency factor expression. FACT binds to gene-distal cis-regulatory elements (promoters and enhancers) and regulates both ncRNA transcription and nucleosome occupancy at these regulatory locations to permit appropriate expression of mRNAs. When FACT is depleted through IAA treatment, nucleosome occupancy at cis-regulatory elements is increased and mRNA expression is altered. These changes result in a loss of pluripotency and initiation of irregular differentiation across all 3 germ layers. OSN = OCT4, SOX2, and/or NANOG. Created with Biorender.com

The role for FACT in pluripotent cells has drawn recent interest but remained mechanistically unclear. Here, we provide a comprehensive analysis of FACT function in murine ES cells and propose that FACT regulates pluripotency, in part, through maintenance of master pluripotency regulators themselves. Specifically, we find that FACT both activates and represses transcription at promoters and gene-distal regulatory regions (Figs. 3 and 5; Table 3). SPT16 binds over many genes (Fig. 2), with enriched binding over FACT-regulated genes compared with those for which transcription is not FACT-regulated (Additional File 1: Fig. S5A-C). However, not all genes differentially expressed were bound by SPT16 (Additional File 1: Fig. S5A-F), suggesting both direct and indirect gene regulation by FACT. To explore a direct, gene-distal regulatory mechanism, we also identify SPT16 binding over gene-distal regions, including over putative enhancers and OSN-bound locations (Fig. 4), where FACT loss also results in altered transcription of ncRNAs (Fig. 5). Finally, we find that SPT16 regulates nucleosome occupancy at both promoter and putative enhancer locations, where SPT16 depletion leads to increased nucleosome occupancy and decreased chromatin accessibility (Fig. 6). Therefore, our studies suggest FACT performs dual roles in transcriptional regulation: facilitation of pluripotency through both coding and non-coding pluripotency-promoting elements and repression of differentiation-promoting elements. Based on these data, we propose a model for FACT activity in ES cells (Fig. 7).

Intriguingly, FACT’s role at gene-distal regulatory elements seems to mirror its role at genic regions, preventing nucleosome occupancy and maintaining gene expression when necessary, while nucleosomes are reconstructed and gene expression is limited at other times. The classes of genes regulated by FACT are not limited to pluripotency genes, however, as pathway analysis identified many distinct pathways among the most enriched for each class of RNA (Fig. 5C, E). Given the extensive non-coding transcription that arises from gene-distal regulatory elements [90], the act of transcription by RNAPII may be the driving force behind increased chromatin accessibility at transcribed regions upon FACT depletion.

### Ideas and speculation

It is tempting to speculate that FACT must maintain accessible chromatin to permit interaction by the master regulators of pluripotency themselves; however, established pioneering activity by OCT4 and SOX2 suggests that the master regulators are not entirely dependent on FACT action [91–94]. FACT depletion has been shown to redistribute histone marks in *D. melanogaster* and *S. cerevisiae*; therefore, disruption of pluripotency-relevant histone marks may be one mechanism through which pluripotency maintenance is affected in FACT-depleted cells [38, 60, 61, 95–97]. This shuffling of histone modifications may then disrupt recruitment of factors that maintain gene expression by sensing histone marks. This disrupted factor recruitment and retention may explain many reductions in transcription following FACT depletion. As FACT binding correlates with CHD1 binding (in *S. cerevisiae*) and gene expression and FACT may remove CHD1 from partially unraveled nucleosomes [47, 72, 98, 99], CHD1 may also become trapped on chromatin without FACT-dependent displacement, thereby reducing expression of target genes.

RNAPII pausing is a phenomenon that occurs at the promoters of coding genes, eRNAs, and PROMPTs [38, 100, 101]. As FACT has been shown to maintain pausing of RNAPII at coding promoters [38], a plausible model emerges through which FACT represses transcription from these regions by maintaining RNAPII pausing to silence improper transcription. This RNAPII pausing-mediated silencing may be the mechanism through which FACT prevents changes in cellular identity (i.e., reprogramming to iPSCs from fibroblasts) [38, 44, 45, 47].

As many groups have suggested, the act of transcription by RNAPII itself may be responsible for destabilization of nucleosomes, creating a genomic conflict for FACT to resolve [26, 37, 72, 81, 98]. With FACT depleted, this nucleosome destabilization likely compounds issues created by failure to maintain RNAPII pausing; it is possible that this combination of genome destabilization and failure to reassemble is responsible for the vast majority of derepressed transcription following FACT depletion. This model is further strengthened by a lack of FACT binding at putative silencers (Additional File 1: Fig. S5G-H); as silencers are unbound by FACT, we did not expect to see altered transcription from silencers upon FACT depletion. Such action would align with a model proposed by Formosa and Winston, wherein cellular FACT dependency results from chromatin disruption and tolerance of DNA packaging defects within the cell [37]. This working model is consistent with the current consensus of the field, as well as emerging studies examining FACT-mediated histone recycling pre- and post-transcription by RNAPII [39]. Together, the work presented in this study provides a preliminary mechanistic understanding for the role of FACT in mammalian pluripotent systems.

## Conclusions

FACT is an important histone chaperone that was previously shown to be specifically required in pluripotent cells. We sought to understand the mechanism through which FACT may contribute to pluripotency. To that end, we generated ES cell lines with degron-tagged SPT16 and found that rapid SPT16 depletion indeed leads to differentiation. Further investigation found that SPT16 loss results in nucleosome invasion into sites of SPT16 binding, which strongly overlaps OSN gene-distal binding sites. Together, these changes in nucleosome binding and chromatin accessibility result in altered mRNA and ncRNA transcription. We propose that SPT16 is required to maintain open chromatin accessibility, OSN binding, and the gene regulatory network responsible for maintaining stem cell identity.

## Methods

### Materials availability

Plasmids and cell lines generated in this study are available on request. All resources generated in this study must be acquired via a Material Transfer Agreement (MTA) granted by the University of Pittsburgh. A complete list of resources can be found in Table S2.

### Cell lines

Murine embryonic stem cells were derived from E14 [102]. Male E14 murine embryonic stem cells were grown in feeder-free conditions on 10-cm plates gelatinized with 0.2% porcine skin gelatin type A (Sigma) at 37 °C and 5% CO_2_. Cells were cultured under LIF + 2i conditions in Dulbecco’s modified Eagle’s medium (Gibco), supplemented with 10% fetal bovine serum (Sigma, 18N103), 0.129 mM 2-mercaptoethanol (Acros Organics), 2 mM glutamine (Gibco), 1X nonessential amino acids (Gibco), 1000U/mL Leukemia Inhibitory Factor (LIF), 3 µM CHIR99021 GSK inhibitor (p212121), and 1 µM PD0325091 MEK inhibitor (p212121). Cells were passaged every 48 h using trypsin (Gibco) and split at a ratio of ~ 1:8 with fresh medium. Routine anti-mycoplasma cleaning was conducted (LookOut DNA Erase spray, Sigma) and cell lines were screened by PCR to confirm no mycoplasma presence.

### Auxin-inducible degradation

Cell lines were constructed in an E14 murine ES cell line with osTIR1 previously integrated into the genome at the *Rosa26* locus. SPT16 was C-terminally tagged using a 39 amino acid mini-AID construct also containing a 3xV5 epitope tag [103–106]. Two homozygous isolated clones were generated using CRISPR-mediated homologous recombination with Hygromycin B drug selection and confirmed by PCR and Sanger sequencing. Cells were depleted of AID-tagged SPT16 protein by addition of 500 µM 3-Indole Acetic Acid (IAA, Sigma) dissolved in 100% EtOH and pre-mixed in fresh medium. Cells were incubated with IAA or 0.1% EtOH (vehicle) for 3, 6, 12, 18, 24, or 48 h to deplete the FACT complex and confirmed by Western blotting. Cells were cultured on 10-cm plates undisturbed for 48 h prior to AID depletion, ensuring that relevant effects are not due to passaging-related disturbances.

### Alkaline phosphatase staining

Cells were treated with EtOH or IAA as described above, with alkaline phosphatase staining after 6, 12, 18, 24 and 48 h. Treated cells were washed twice in 1 × Dulbecco’s phosphate-buffered saline (DPBS, Gibco) and crosslinked in 1% formaldehyde (Fisher) in DPBS for 5 min at room temperature. Crosslinking was quenched with 500 mM glycine, and cells were washed twice in 1 × DPBS. Cells were stained with Vector Red Alkaline Phosphatase Staining Kit (Vector Labs) per the manufacturer’s instructions in a 200 mM Tris–Cl buffer, pH 8.4. Eight mL working solution was added to each 10-cm plate and incubated in the dark for 30 min before being washed with DPBS and imaged. Alkaline phosphatase staining was quantified using FIJI [70]. Red channels were isolated from individual images, converted to a binary image and quantified by integrated density.

### Immunocytochemistry

Cells were treated with EtOH or IAA as described above. At the indicated depletion timepoints, cells were crosslinked in 4% formaldehyde in DPBS and permeabilized with 0.2% Triton X-100 (Fisher). Permeabilized cells were washed, blocked for 1 h at room temperature in 5% bovine serum albumin in DPBS, and washed. Cells were incubated in anti-OCT4 primary antibody (ReproCELL 09–0023, lot J17070000000001) overnight at a 1:500 dilution in 1% BSA-DPBS. Cells were washed and incubated in fluorophore-conjugated secondary antibody at a 1:500 dilution for 1 h at room temperature (Vector Labs lot ZJ0214). Cells were washed and incubated in 2 µg/mL DAPI (StemCell Technologies 75004) in DPBS for 10 min to stain nuclei, washed, and imaged on a fluorescent microscope (Nikon Eclipse Ts2). OCT4 ICC was quantified using FIJI [70]. Green channels were isolated from individual images, converted to a binary image and quantified by integrated density.

### Western blotting

Western blotting was performed using monoclonal antibodies against the V5 epitope (mouse; Invitrogen 46–0705, lot 1923773), SSRP1 (mouse; BioLegend 609702, lot B280320), OCT4 antibody (rabbit; Invitrogen 701756, lot 2263537), and beta-actin (mouse; Sigma A1978, lot 037M4782V). Secondary antibody incubations were performed with goat polyclonal antibodies against either rabbit or mouse IgG, (Bio-Rad 170–6515, lot #64149722, BioRad 170–6516, lot #64147779). Crude protein extractions were performed using RIPA buffer (150 mM NaCl, 1% IPEGAL CA-630, 0.5% sodium deoxycholate, 0.1% sodium dodecyl sulfate, and 25 mM Tris–Cl, pH 7.4) with freshly added protease inhibitors (Thermo Fisher) and flash-frozen immediately after extraction. Samples were quantitated using the Pierce BCA Protein Assay kit (Thermo Fisher). Twenty µg were diluted in RIPA buffer with 10 mM dithiothreitol (DTT) and Laemmeli sample buffer before being loaded on 7.5% Tris-acrylamide gels for Western blotting. Proteins were transferred to nitrocellulose membranes (BioTrace) via a Criterion tank blotter (BioRad) at 100 V for 1 h and stained with 0.5% Ponceau S (Sigma) in 1% acetic acid to confirm proper transfer. Membranes were blocked in 5% milk in PBST prior to overnight primary antibody incubation at 4°C. Membranes were then washed and incubated in secondary antibody (Bio-Rad) for 1 h at room temperature, washed, and developed with SuperSignal West Pico chemiluminescent reagent (Thermo Fisher) for 5 min at room temperature. Raw, unedited blot images can be found in Additional Files 3.

### CUT&RUN

CUT&RUN was performed as described [71, 107–109], using recombinant Protein A/Protein G-MNase (pA/G-MN) [110]. Briefly, 100,000 nuclei were isolated from cell populations using a hypotonic buffer (20 mM HEPES–KOH, pH 7.9, 10 mM KCl, 0.5 mM spermidine, 0.1% Triton X-100, 20% glycerol, freshly added protease inhibitors) and bound to lectin-coated concanavalin A magnetic beads (200 µL bead slurry per 500,000 nuclei) (Polysciences). Immobilized nuclei were chelated with blocking buffer (20 mM HEPES, pH 7.5, 150 mM NaCl, 0.5 mM spermidine, 0.1% BSA, 2 mM EDTA, fresh protease inhibitors) and washed in wash buffer (20 mM HEPES, pH 7.5, 150 mM NaCl, 0.5 mM spermidine, 0.1% BSA, fresh protease inhibitors). Nuclei were incubated in wash buffer containing primary antibody (anti-V5 mouse monoclonal, Invitrogen 46–0705, lot 1923773) for 1 h at room temperature with rotation, followed by incubation in wash buffer containing recombinant pA/G-MN for 30 min at room temperature with rotation. Controls lacking a primary antibody were subjected to the same conditions but incubated in wash buffer without antibody prior to incubation with pA/G-MN. Samples were equilibrated to 0 °C, and 3 mM CaCl_2_ was added to activate pA/G-MN cleavage. After suboptimal digestion for 15 min, digestion was chelated with 20 mM EDTA and 4 mM EGTA, and 1.5 pg MNase-digested *S. cerevisiae* mononucleosomes were added as a spike-in control. Genomic fragments were released after an RNase A treatment. After separating released fragments through centrifugation, fragments isolated were used as input for a library build consisting of end repair and adenylation, NEBNext stem-loop adapter ligation, and subsequent purification with AMPure XP beads (Agencourt). Barcoded fragments were then amplified by 14 cycles of high-fidelity PCR and purified using AMPure XP. Libraries were pooled and sequenced on an Illumina NextSeq500 to a depth of ~ 10 million mapped reads.

### CUT&RUN data analysis

Paired-end fastq files were trimmed to 25 bp and mapped to the mm10 genome with bowtie2 (options -q -N 1 -X 1000) [111]. Mapped reads were duplicate-filtered using Picard [112] and filtered for mapping quality (MAPQ ≥ 10) using SAMtools [113]. Size classes corresponding to FACT footprints (< 120 bp) were generated using SAMTools [113]. Reads were converted to bigWig files using deepTools with the TPM-related read normalization RPGC (options -bs 1 –normalizeUsing RPGC, –effectiveGenomeSize 2862010578) [114], with common sequencing read contaminants filtered out according to ENCODE blacklisted sites for mm10. Heatmaps were generated using deepTools computeMatrix (options -a 2000 -b 2000 -bs 20 –missingDataAsZero) and plotHeatmap [114]. Peaks were called from CUT&RUN data using SEACR, a CUT&RUN-specific peak-calling algorithm with relaxed stringency and controls lacking primary antibody used in lieu of input data [110]. Motifs were then called from these peaks using HOMER with default settings [74]. Pathway analysis was performed on peaks present in at least 2/4 SPT16-V5 CUT&RUN experiments using HOMER and the WikiPathways database, then plotted in GraphPad Prism 9, with the *y*-axis representing rank of enrichment [74]. To ensure that only direct effects were captured, we limited our analysis to genes with an SPT16-V5 peak within 1000 bp of the gene TSS. To control for ES cell-specific background noise, we kept only genes with a DESeq2 baseMean ≥ 1 (TT-seq) and used a background gene list of all genes with a baseMean ≥ 1 in ES cells. The Benjamini–Hochberg correction was applied to *p*-values with an FDR of 0.05 to correct for multiple testing.

One-dimensional heatmap matrices generated using deepTools computeMatrix as above were averaged by position relative to reference point using plotProfile with the option –outFileNameMatrix. Average position scores per technical replicate were then averaged together and translated to colorimetric scores using ggplot2 [115].

### Transient transcriptome sequencing

TT-seq was performed using a modified method [79, 116–118]. Five mM 4-thiouridine (4sU; Carbosynth T4509) was dissolved in 100% DMSO (Fisher). For IAA-washout experiments, cells were depleted of SPT16 by treatment with EtOH or 500 µM IAA for 12 h, then medium was replaced with fresh, untreated medium for 24 h. Following protein depletion as above, cells were washed with 1 × DPBS (Corning), resuspended in medium containing 500 µM 4sU, and incubated at 37°C and 5% CO_2_ for 5 min to label nascent transcripts. After washing cells with 1 × DPBS, RNA was extracted with TRIzol and fragmented using a Bioruptor Pico for one cycle at high power. Thiol-specific biotinylation of 100 µg of total RNA was carried out using 10x biotinylation buffer (100 mM Tris–Cl, pH 7.4, 10 mM ethylenediaminetetraacetic acid) and EZ-Link Biotin-HPDP (Pierce 21341) dissolved in dimethylformamide (Fisher) at 1 mg/mL. Biotinylation was carried out for 2 h away from light with 1000 rpm shaking at 37°C. RNA was extracted with chloroform and precipitated using NaCl and isopropanol. Labeled RNA was separated from unlabeled RNA via a streptavidin C1 bead-based pulldown (DynaBeads, Thermo Fisher). In brief, beads were washed in bulk in 1 mL of 0.1N NaOH with 50 mM NaCl, resuspended in binding buffer (10 mM Tris–Cl, pH 7.4, 0.3M NaCl, 1% Triton X-100) and bound to RNA for 20 min at room temperature with rotation. Beads bound to labeled RNA were washed twice with high salt wash buffer (5 mM Tris–Cl, pH 7.4, 2M NaCl, 1% Triton X-100), twice with binding buffer, and once in low salt wash buffer (5 mM Tris–Cl, pH 7.4., 1% Triton X-100). Nascent RNA was recovered from beads using two elutions with fresh 100 mM dithiothreitol at 65°C for 5 min with 1000 rpm shaking. Recovered nascent RNA was then extracted with PCI and chloroform, and then isopropanol precipitated.

Strand-specific nascent RNA-seq libraries were built using the NEBNext Ultra II Directional Library kit, with the following modifications: 200 ng of fragmented RNA was used as input for ribosomal RNA removal via antisense tiling oligonucleotides and digestion with thermostable RNase H (MCLabs) [119, 120]. rRNA-depleted RNA samples were treated with Turbo DNase (Thermo Fisher) and purified by silica column (Zymo RNA Clean & Concentrator). RNA was fragmented at 94°C for 5 min and subsequently used as input for cDNA synthesis and strand-specific library building, per the manufacturer’s protocol. Libraries were pooled and sequenced via Illumina NextSeq500 or NextSeq2000 to a depth of ~ 40 million mapped reads.

### TT-seq data analysis

Paired-end fastq files were trimmed and filtered using Trim Galore [121], then aligned to the mm10 mouse genome using STAR (options –outSAMtype SAM –outFilterMismatchNoverReadLmax 0.02 –outFilterMultimapNmax 1). Feature counts were generated using subread featureCounts (options -s 2 -p -B) for genes, PROMPTs, DHSs, and putative eRNAs based on genomic coordinates (see next paragraph) [122]. No filtering for baseline expression was applied due to the sensitivity of TT-seq in detecting lowly expressed transcripts. To visualize TT-seq data, bigwigs were generated using deepTools with TPM read normalization (options -bs 1 –normalizeUsing BPM) [114]. Reads were imported to R and downstream analysis was conducted using DESeq2 [123]. Differentially expressed transcripts were plotted using EnhancedVolcano [124]. Pathway (GO-term) analysis was performed on significantly up- and downregulated genes separately using HOMER with the WikiPathways database [74, 125]. Only transcripts with a DESeq2 baseMean value of ≥ 1 were used to filter out lowly transcribed regions. Significance was defined as DESeq2 adjusted *p*-value < 0.05. Top five enriched categories were plotted in GraphPad Prism 9 against − log_10_ *p*-value, along with pluripotency-related categories added from among the top 50 most enriched pathways. *Y*-axes indicate pathway enrichment ranking. For downstream analyses, we generated GTF and bed files of Gencode mm10 vM25 genes, sorted by nascent transcription in all control (Untagged, 0 h, and EtOH-treated) samples, pooled together.

Non-coding transcripts were identified by removing all transcription start sites within 1 kb of annotated mm10 coding genes from the previously described TSS-distal DNaseI hypersensitive sites (GSM1014154) [83–85]. PROMPTs were called by genomic location (within 1 kb of an annotated mm10 TSS and divergently transcribed to the TSS). ncRNAs were assigned to the closest coding gene and pathway analysis was conducted as above. Putative enhancers were defined as overlapping a DHS, as well as the presence of either H3K27ac or H3K4me1, according to ChIP-seq data from ENCODE [83–85].

### Traditional RNA sequencing

RNA-seq was performed as previously described [126]. Following protein depletion as above, cells were washed with 1 × DPBS (Corning). After washing, RNA was extracted with TRIzol per the manufacturer’s instructions and purified by chloroform extraction and isopropanol precipitation. Extracted RNA was flash-frozen in liquid nitrogen and stored until use.

One hundred micrograms of RNA was used as input for ribosomal RNA removal via antisense tiling oligonucleotides and digestion with thermostable RNase H (MCLabs) [119, 120]. rRNA-depleted RNA samples were treated with Turbo DNase (Thermo Fisher) and purified by silica column (Zymo RNA Clean & Concentrator). Purified rRNA-depleted steady-state RNA samples were used as input for strand-specific RNA-seq library builds, using the NEBNext Ultra II Directional Library kit. RNA was fragmented at 94°C for 5 min and subsequently used as input for cDNA synthesis and strand-specific library building, per the manufacturer’s protocol. Libraries were pooled and sequenced via Illumina NextSeq2000 to a depth of ~ 20 million mapped reads.

### RNA-seq data analysis

Paired-end fastq files were trimmed and filtered using Trim Galore [121], then aligned to the mm10 mouse genome using STAR (options –outSAMtype SAM –outFilterMismatchNoverReadLmax 0.02 –outFilterMultimapNmax 1). Feature counts were generated using subread featureCounts (options -s 2 -p -B) [122]. To visualize RNA-seq data, bigwigs were generated using deepTools with TPM read normalization (options -bs 1 –normalizeUsing BPM) [114]. Reads were imported to R and downstream analysis was conducted using DESeq2 [123]. Differential gene expression was plotted using EnhancedVolcano [124]. Pathway (GO-term) analysis was performed on significantly up- and downregulated genes separately using HOMER with the WikiPathways database [74, 125]. Only genes with a DESeq2 baseMean value of ≥ 1 were used to filter out un- or lowly expressed genes. Significance was defined as DESeq2 adjusted *p*-value < 0.05. Top five enriched categories were plotted in GraphPad Prism 9 against − log_10_ *p*-value, along with pluripotency-related categories added from among the top 50 most enriched pathways. *Y*-axes indicate pathway enrichment ranking.

### Reverse transcription and quantitative PCR (RT-qPCR)

RT-qPCR was performed as previously described [127]. Briefly, RNA was extracted from cells using TRIzol following treatment with either IAA or EtOH for 0, 3, and 6 h. One microgram of RNA was used as input for reverse transcription, and quantitative PCR was performed using 5 µM PCR primers targeting the gene of interest with KAPA SYBR green master mix. Technical replicates shown represent the average of three individual qPCR reactions for each treatment/target/condition group. Error bars shown represent the standard deviation of two replicates for each combination.

### Assay for transposase-accessible chromatin sequencing (ATAC-seq)

Omni-ATAC-seq was performed as previously described [128]. Briefly, cells were depleted of SPT16 using a treatment with EtOH (vehicle) or 500 µM IAA for 0, 3, 6, 12, or 24 h. For IAA-washout experiments, cells were depleted of SPT16 by treatment with EtOH or 500 µM IAA for 12 h, then medium was replaced with fresh, untreated medium for 24 h. Nuclei were extracted from 60,000 cells as described for CUT&RUN and flash-frozen until use. Frozen nuclei were resuspended in transposition mix containing 1 × TD buffer (10 mM Tris pH 7.6, 5 mM MgCl_2_, 10% dimethylformamide), DPBS, 0.1% Tween-20, 1% digitonin, and 4 µL Tn5 transposome (Diagenode) per reaction. Samples were incubated at 37 °C for 30 min with 1000 rpm shaking. Transposed DNA was purified using a Clean and Concentrator kit (Zymo) per the manufacturer’s instructions. Samples were amplified for 5 cycles of high-fidelity PCR (KAPA), then held on ice and assessed via qPCR (KAPA SYBR Green). Samples were then returned to the thermocycler for as many cycles as needed to reach 1/3 qPCR saturation (~ 10 total cycles). Amplified libraries were gel-extracted between 150 and 650 bp and sequenced via Illumina NextSeq2000 to a sequencing depth of ~ 50 million mapped reads.

### ATAC-seq data analysis

Paired-end fastq files were trimmed to 25 bp and mapped to the mm10 genome with Bowtie 2 (using the options –very-sensitive –dovetail -q -N 1 -X 1000) [111]. Mapped reads were duplicate-filtered using Picard [112] and filtered for mapping quality (MAPQ ≥ 10) using SAMtools [113]. Reads were separated into size classes of 1–100 bp (factor binding) and 180–247 bp (mononucleosomal fragments) using an awk command. Size-selected reads were converted to bigWig files using deepTools with the TPM-related read normalization RPGC (options -bs 1 –normalizeUsing RPGC, –effectiveGenomeSize 2308125349 –ignoreForNormalization chrM -e) [114]. Differential bigwigs were generated using deepTools bigwigCompare (-bs 10) [114]. Heatmaps were generated using deepTools computeMatrix (options –referencePoint TSS -a 2000 -b 2000 -bs 20 –missingDataAsZero) and plotHeatmap, based on the 1–100 size class [114]. Differences in accessibility were plotted by generating matrices in deepTools as above. Where indicated, data were clustered using *k*-means clustering.

In parallel, we analyzed our ATAC-seq data using PEPATAC, via the standard analysis pipeline [129]. PEPATAC was used to quality-check the ATAC-seq datasets, ensuring that all replicates had TSS enrichment scores of > 10. We then called consensus peaks from combined ATAC-seq replicates, taking only peaks that were present in (*n*/2) + 1 samples, regardless of condition. Over these consensus peaks, we identified differential enrichment using HOMER getDifferentialPeaks.pl and performed pairwise Pearson correlations using deepTools under default parameters [74, 114].

### Micrococcal nuclease sequencing (MNase-seq)

MNase-seq was performed as previously described [127, 130, 131]. In brief, cells were depleted of SPT16 using a 24-h treatment with EtOH (vehicle) or 500 M IAA. Five million cells were collected, crosslinked using 1% formaldehyde for 15 min at RT, and quenched with 500 mM glycine. Cells were lysed in hypotonic buffer (10 mM Tris–Cl, pH 7.5, 10 mM NaCl, 2 mM MgCl_2_, 0.5% NP-40, 0.3 mM CaCl_2_, and 1 × protease inhibitors) and subjected to 5 min of digestion with MNase (TaKaRa) at 37°C before chelation with EDTA and EGTA. Samples were treated with RNase A (Thermo Fisher) for 40 min at 37°C and 1000 rpm constant shaking in a thermomixer. Crosslinks were reversed overnight at 55°C and chromatin was digested with Proteinase K, then used as input for a paired-end library build.

One microgram input DNA was treated with Quick CIP (NEB) for 30 min and heat-inactivated. End repair was then performed using T4 DNA Polymerase (NEB), T4 Polynucleotide Kinase (NEB), and Klenow DNA Polymerase (NEB) simultaneously. A-overhangs were added to sequences via treatment with Klenow Polymerase without exonuclease activity, and Illumina paired-end TruSeq adapters were added using Quick Ligase (NEB). Barcoded DNA was purified using AMPure XP beads (Agencourt) and amplified by high-fidelity PCR (KAPA). Completed libraries were subjected to silica column purification (Zymo DNA Clean & Concentrator) and sequenced via Illumina NextSeq500 to a sequencing depth of ~ 50 million mapped reads.

### MNase-seq data analysis

Paired-end fastq files were trimmed to 25 bp and mapped to the mm10 genome with bowtie2 (using the options -q -N 1 -X 1000) [111]. Mapped reads were duplicate-filtered using Picard [112] and filtered for mapping quality (MAPQ ≥ 10) using SAMtools [113]. Reads were then sorted into nucleosome-sized (135–165 bp) fragments using SAMtools [113]. Nucleosome-sized reads were converted to bigWig files using deepTools with the TPM-related read normalization RPGC (options -bs 1 -e –normalizeUsing RPGC, –effectiveGenomeSize 2862010578), with common sequencing read contaminants filtered out according to ENCODE blacklisted sites for mm10 [114]. Differential bigwigs were generated using deepTools bigwigCompare (default options) [114]. Heatmaps were generated using deepTools computeMatrix (options –referencePoint TSS -a 2000 -b 2000 -bs 20 –missingDataAsZero) and plotHeatmap and plotProfile [114]. Differences in nucleosome occupancy were plotted by generating matrices in deepTools as above. Metaplots of MNase-seq data in Figs. 6 and S7 include standard error shaded around the plotted line (mean).

### Statistics

Statistical details for each experiment shown can be found in the accompanying figure legends. Where indicated, “n” designates independent technical replicates for the same biological sample, while biological replicates are referred to as “clone 1” and “clone 2” to differentiate between independently targeted cell lines. Statistical tests were used in TT-seq analyses as per the default parameters for DESeq2, with a correction applied to minimize fold change of lowly expressed transcripts (LFCshrink) [123, 132]. Differences in altered transcription were analyzed for significance using Friedman tests and two-way ANOVAs, with corrections for multiple comparisons performed using Dunn’s and Dunnett’s tests, respectively. Motif analyses (HOMER) and peak-calling (SEACR and HOMER for CUT&RUN and ChIP-seq datasets, respectively) were performed using default program parameters [73, 74]. Any error bars shown represent one standard deviation in both directions. Standard error was calculated via deepTools plotProfile for MNase-seq metaplots generated in Figs. 6 and S6. Significance was defined as a *p*-value < 0.05 by the respective test performed (indicated with “*”). For RNA-seq and TT-seq analyses, adjusted *p*-values were used for significance cutoffs. No data or subjects were excluded from this study. Average values for CUT&RUN, ChIP-seq, and MNase-seq datasets were determined by computing the mean of coverage at each base pair throughout the genome between replicates. Merged replicates indicates mean of read-coverage normalized tracks generated for each individual replicate. Overlaps between individual DNA-binding profiling experiments (ChIP-seq and CUT&RUN) were assessed via Fisher’s exact tests, performed using the Bedtools “fisher” command, comparing peaks called from the individual datasets [133]. Altered chromatin accessibility and nucleosome occupancy trends were analyzed for significance using Friedman tests and corrected for multiple comparisons using Dunn’s test. Unless otherwise noted, all statistical comparisons were performed in GraphPad PRISM 9. Correlative analyses were performed to compare replicates between NGS datasets for similarity and are displayed in Additional File 1: Fig. S11.

### Supplementary Information

**Additional file 1: Fig. S1.** Characterization of SPT16-V5-AID cell lines. **Fig. S2.** ES cell culture conditions show similar trends of SPT16-V5 binding and pluripotency factor binding. **Fig. S3.** Characterization of transcriptomic effects of SPT16 depletion and FACT interactions with pluripotency factors. **Fig. S4.** Sankey plots depicting altered transcripts at 3, 6, 12, and 24 h of treatment. **Fig. S5.** SPT16-V5 binding is enriched at promoters of FACT-regulated genes but not at putative silencers. **Fig. S6.** FACT depletion reduces transcription at superenhancers. **Fig. S7.** FACT depletion disrupts nucleosome positioning and chromatin accessibility at regulatory regions. **Fig. S8.** FACT depletion disrupts chromatin accessibility at regulatory regions. **Fig. S9.** Washout of IAA does not restore chromatin accessibility changes induced by 12-h FACT depletion. **Fig. S10.** FACT depletion disrupts nucleosome positioning at regulatory regions. **Fig. S11.** Validation of NGS datasets. **Table S2.** Key reagents, cell lines, and datasets used in this work.

**Additional file 2: Table S1.** Comparison of selected pluripotency and differentiation factor expression and transcription over time. DESeq2-calculated log_2_-fold changes in transcription (TT-seq) and expression (RNA-seq) of pluripotency factors and differentiation factors associated with ectoderm, endoderm, and mesoderm are displayed alongside adjusted p-values for all transcriptomic experiments performed, including TT-seq after IAA treatment (0-, 3-, 6-, 12-, 24-, and 12-h+24-h washout) and RNA-seq after 24-h IAA treatment.

**Additional file 3.** Unedited, uncropped Western blots displayed in Figs. 1 and S1. Blots are presented in the following order: Fig. 1A (V5-SPT16); Fig. 1A (Actin); Additional File 1: Fig. S1A (V5-SPT16); Additional File 1: Fig. S1A (Actin); Additional File 1: Fig. S1B (OCT4); Additional File 1: Fig. S1B (SSRP1 and Actin); Additional File 1: Fig. S1C (V5-SPT16); Additional File 1: Fig. S1C (SSRP1); Additional File 1: Fig. S1C (Actin).

## Data Availability

This paper analyzes existing, publicly available data housed in the NCBI Gene Expression Omnibus (GEO) and the Sequence Read Archive (SRA). The accession numbers for the datasets are listed throughout the manuscript and can all be found in Additional File 1: Table S2. Unedited raw sequencing reads and processed bigwig files generated during this study have been deposited in NCBI GEO under the accession number GSE181624. Any additional information required regarding the data reported in this paper is available from the lead contact upon request.

## Abbreviations

IAA: 3-Indole acetic acid (Auxin)
4sU: 4-Thiouridine
AID: Auxin-inducible degradation
AP: Alkaline phosphatase
ATAC-seq: Assay for transposase-accessible chromatin
ChIP-seq: Chromatin immunoprecipitation and deep sequencing
CUT&RUN: Cleavage Under Targets & Release Using Nuclease
DHS: TSS-distal DNase I Hypersensitive Sites
eRNA: Enhancer RNA
ES cell: Embryonic stem cell
FACT: Facilitates Chromatin Transcription Complex
GO: Gene Ontology
MNase: Micrococcal nuclease
MNase-seq: Micrococcal nuclease digestion paired with deep sequencing
mRNA: Messenger RNA
ncRNA: Non-coding RNA
OSN: OCT4, SOX2, and NANOG
pA/G-MNase: Recombinant Protein A and Protein G fused to Micrococcal Nuclease
PCA: Principal component analysis
PROMPT: Promoter-Upstream Transcript (also known as uaRNA)
RNAPII: RNA polymerase II
RT-qPCR: Reverse transcription and quantitative polymerase chain reaction
TSS: Transcription start site
TT-seq: Transient transcriptome sequencing
uaRNA: Upstream antisense RNA (also known as PROMPT)
