# Supplementary material for: FACT regulates pluripotency through proximal and distal regulation of gene expression in murine embryonic stem cells
Source: BMC Biol. 2023 Aug 4;21:167. doi: 10.1186/s12915-023-01669-0 (PMC10403911; doi:10.1186/s12915-023-01669-0)
Supplement: Supplementary file 1 — Additional file 1: Fig. S1. Characterization of SPT16-V5-AID cell lines. Fig. S2. ES cell culture conditions show similar trends of SPT16-V5 binding and pluripotency factor binding. Fig. S3. Characterization of transcriptomic effects of SPT16 depletion and FACT interactions with pluripotency factors. Fig. S4. Sankey plots depicting altered transcripts at 3, 6, 12, and 24 h of treatment. Fig. S5. SPT16-V5 binding is enriched at promoters of FACT-regulated genes but not at putative silencers. Fig. S6. FACT depletion reduces transcription at superenhancers. Fig. S7. FACT depletion disrupts nucleosome positioning and chromatin accessibility at regulatory regions. Fig. S8. FACT depletion disrupts chromatin accessibility at regulatory regions. Fig. S9. Washout of IAA does not restore chromatin accessibility changes induced by 12-h FACT depletion. Fig. S10. FACT depletion disrupts nucleosome positioning at regulatory regions. Fig. S11. Validation of NGS datasets. Table S2. Key reagents, cell lines, and datasets used in this work. [file 12915_2023_1669_MOESM1_ESM.pdf]

Additional File 1: Figs. S1-S11, Table S2.

Supplemental Figures

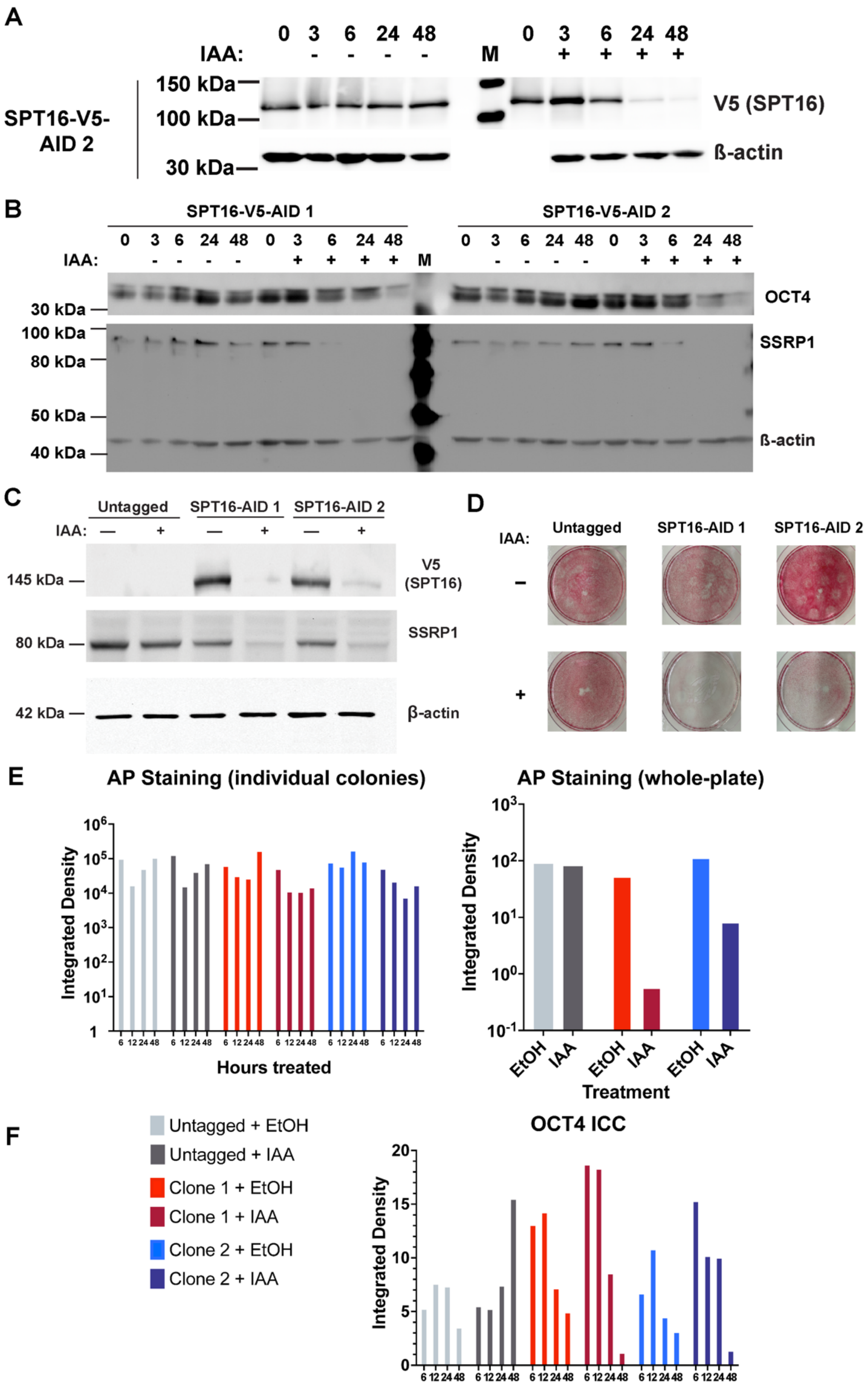

Fig. S1. Characterization of SPT16-V5-AID cell lines. A. Timecourse of IAA treatment for SPT16 depletion in two independently targeted SPT16-V5-AID cell lines. 40 μg total protein

loaded. Top: V5 (targeting SPT16); bottom: β-actin (run on separate blots). IAA – indicates vehicle control (EtOH). B. Timecourse of OCT4 (top), SSRP1, reprobed for β-actin (bottom) protein levels following IAA treatment to deplete SPT16. 40 μg total protein loaded. IAA – indicates vehicle control (EtOH). C. Western blot depicting protein after 24-hour treatment, probed for V5-SPT16 (top), SSRP1 (middle) and β-actin (bottom). 40 μg total protein loaded. IAA – indicates vehicle control (EtOH). D. 10 cm plate images of alkaline phosphatase-stained cells following 24-hour IAA treatment (bottom) or vehicle control (top). E. Quantitation of alkaline phosphatase staining presented in Figure 1C (left) and Figure S1D (right). Red channels were isolated from individual images using FIJI, converted to a binary image and quantified using integrated density. F. Quantitation of OCT4 ICC presented in Figure 1D (right). Green channels were isolated from individual images using FIJI, converted to a binary image and quantified by integrated density.

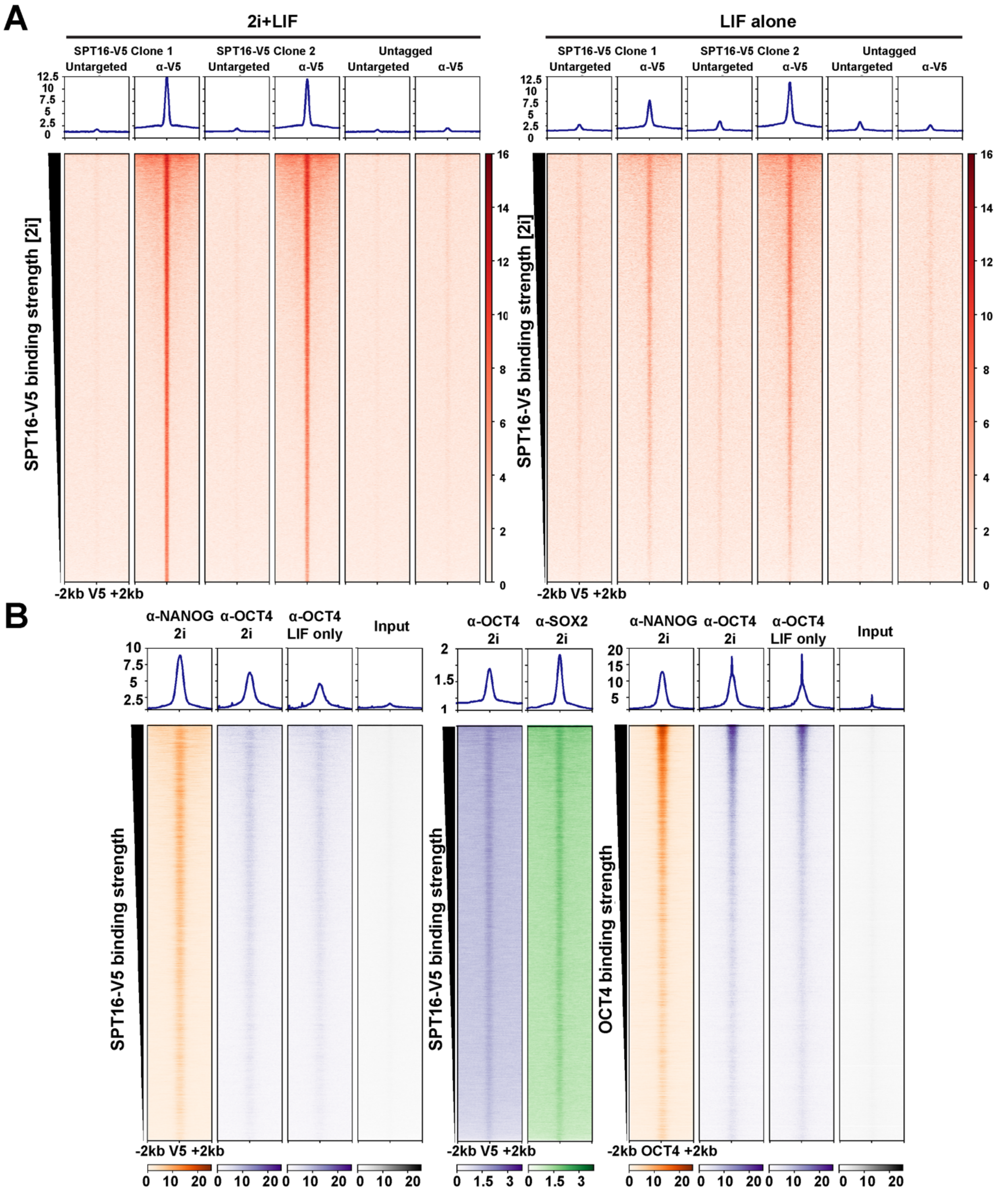

Fig. S2. ES cell culture conditions show similar trends of SPT16-V5 binding and pluripotency factor binding. A. SPT16-V5 CUT&RUN data from ES cells grown in 2i+LIF (left) or LIF alone (right). Merged data from independent technical replicates are displayed together

and are sorted by SPT16-V5 CUT&RUN binding in the 2i-cultured replicates for both heatmaps. n = 3 for untagged cell lines (2i), n = 2 for tagged cell lines (2i); in LIF, n = 2 for untagged cell lines and n = 1 for tagged cell lines. B. Comparison of public ChIP-seq datasets for pluripotency factors in 2i and LIF alone at SPT16-V6 binding sites (left, center) and at OCT4 ChIP-seq peaks from GSE11724. ChIP-seq datasets from GSE56312 and GSE174774.

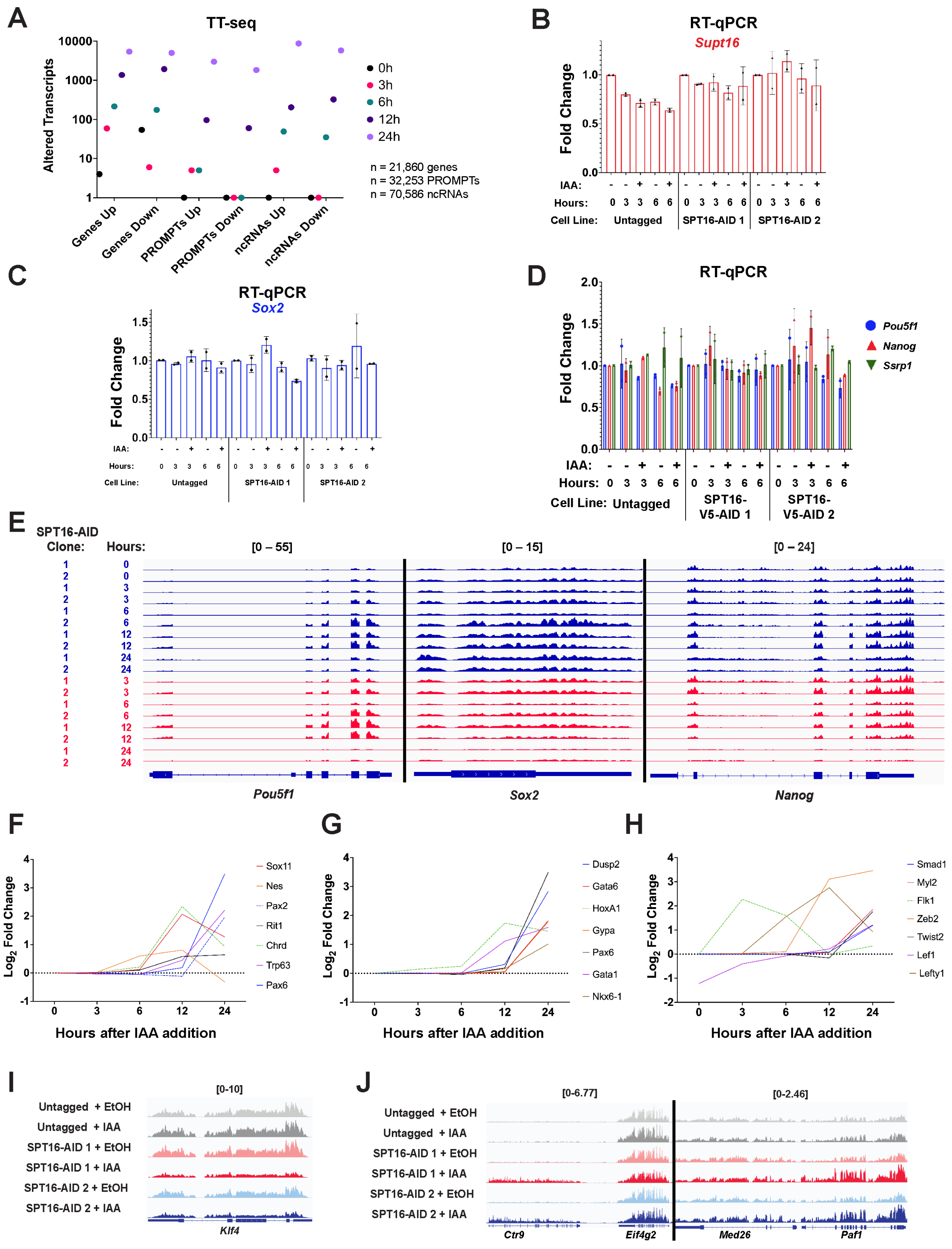

Fig. S3. Characterization of transcriptomic effects of SPT16 depletion and FACT

interactions with pluripotency factors. A. Dot plot depicting the number of differentially expressed genes, Promoter-upstream transcripts (PROMPTs), and ncRNAs transcribed from gene-distal DNaseI hypersensitive sites (DHSs) (DNase-seq from GSM1014154) (Analyzed with DESeq2). Number of transcripts in each category are provided in Table 3. One count was added to each category for plotting. Data were analyzed for significance via a two-way ANOVA and corrected for multiple comparisons using Dunnett's test. Significant differences between transcript class were not identified ($p = 0.3579$, 5.077% of total variation), while each class of transcripts was significantly altered over depletion time ($p < 0.0001$, 77.58% of total variation). B-D. Short-term IAA treatment (3- and 6-hour) for SPT16 depletion followed by RT-qPCR for *Supt16* (B) *Sox2* (C) *Pou5f1*, *Nanog*, and *Ssrp1* (D) mRNA abundances. Fold change calculated using $\Delta\Delta$Ct with normalization to *Pgk1* transcript abundance, where 0h timepoint is set to 1 and other timepoints are made relative. Error bars represent one standard deviation of fold change ($n = 2$ biological replicates). E**.** IGV genome browser tracks depicting nascent transcription at the *Pou5f1* (left), *Sox2* (middle), and *Nanog* (right) genomic loci. Samples were treated with either IAA (red) or vehicle (blue) for the indicated length. 24-hour samples are averaged technical replicates ($n = 3$); all other samples are individual technical replicates. F-H. Expression of differentiation markers over TT-seq timecourse representing early markers of ectoderm (F), endoderm (G) and mesoderm (H) lineages. Significance of altered differentiation factor transcription was analyzed by Friedman tests and corrected for multiple comparisons via Dunn's test ($p = 0.0256$ [F], $p = 0.0002$ [G], $p = 0.0087$ [H]. I. IGV genome browser tracks depicting nascent transcription at the *Klf4* genomic locus following 24 hours of IAA treatment to deplete SPT16. Browser tracks represent merged technical replicates ($n = 3$), while biological replicates are displayed separately. J. As in B but depicting nascent transcription at the *Ctr9* (left) and *Paf1* (right) genomic loci. Technical replicates are averaged ($n = 3$).

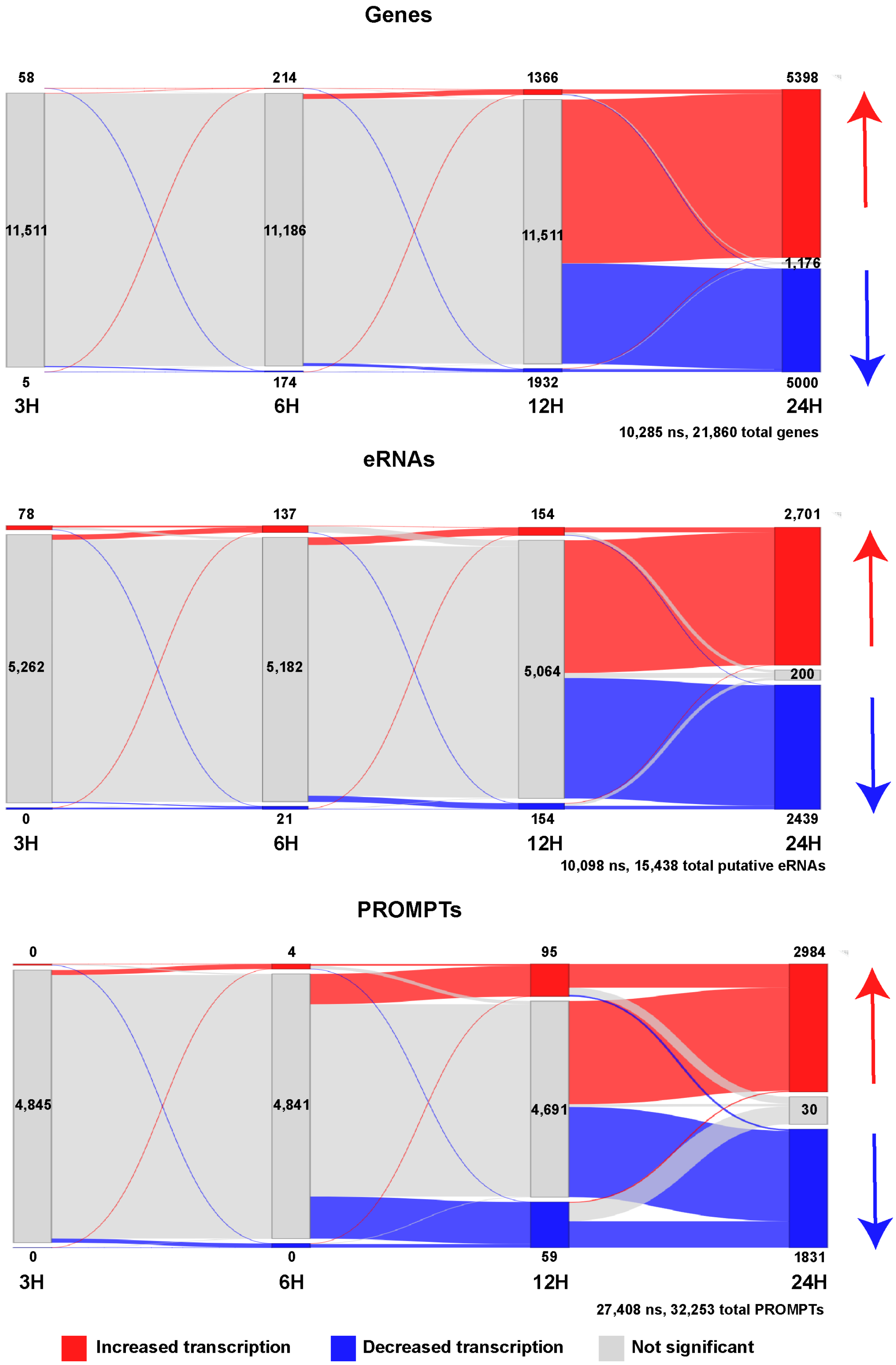

Fig. S4. Sankey plots depicting altered transcripts at 3, 6, 12, and 24 hours of treatment.

Transcripts which never significantly changed were not plotted (ns). Red indicates increased transcripts, while blue indicates decreased transcriptions between timepoints. Each node indicates transcripts in one category at one timepoint, while flows indicate transcript changes between timepoints. Input values were taken from DESeq2 results listed in Table 2. Data were analyzed for significance via a two-way ANOVA and corrected for multiple comparisons using Dunnett's test. Significant differences between transcript class were not identified ($p = 0.3579$, 5.077% of total variation), while the same class of transcripts was significantly altered over depletion time ($p < 0.0001$, 77.58% of total variation).

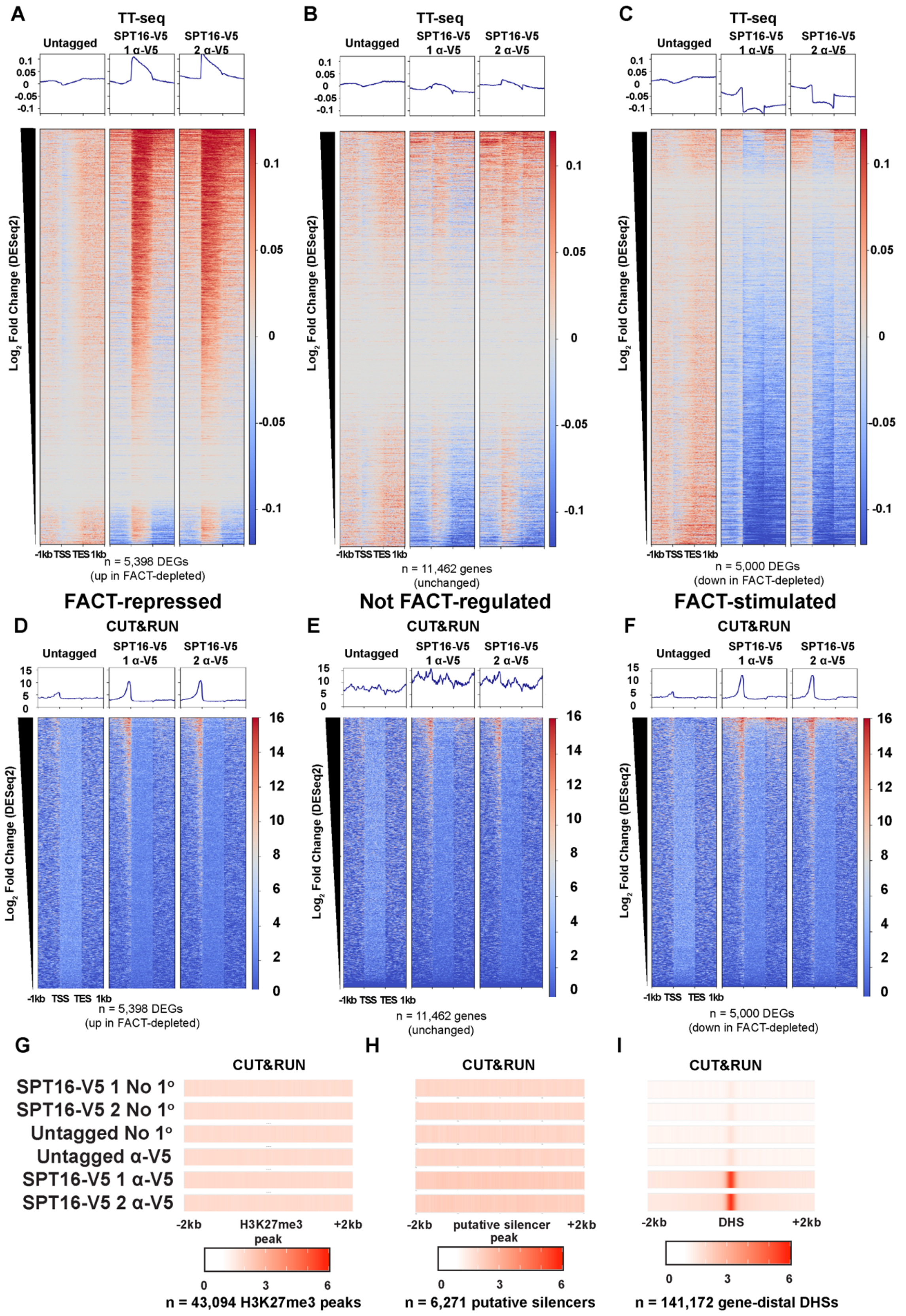

Fig. S5. SPT16-V5 binding is enriched at promoters of FACT-regulated genes but not at

putative silencers. A-C. Nascent transcription following 24 hours of IAA treatment to deplete SPT16. Merged replicates shown as metagene plots, ±1 kb from the start or end site of transcription (n = 3 per plot). Genes sorted by descending $\log_2$ fold change in DESeq2 results. Visualized over genes with significantly increased transcription (adj. $p < 0.05$ and $\log_2$ fold change > 0.75) (A), genes with expression unaffected after FACT depletion (adj. $p > 0.05$ or $|\log_2$ fold change$| < 0.75$) (B), and genes with reduced transcription following FACT depletion (adj. $p < 0.05$, $\log_2$ fold change < -0.75) (C). D-F. SPT16-V5 CUT&RUN visualized over genes classified by transcriptional change after 24 hours of IAA treatment to deplete SPT16. Merged replicates shown as metagene plots, ±1 kb from the start or end site of transcription (n = 3 for untagged, n = 2 for all other samples). Genes sorted by descending $\log_2$ fold change in DESeq2 results. Visualized over significantly increased transcription (D), unchanged transcription (E), or reduced transcription (F) as in A-C. G. SPT16-V5 CUT&RUN data visualized at H3K27me3 ChIP-seq peaks ±2 kb as one-dimensional heatmaps (K27me3 ChIP-seq from GSE123174). Shown as average of technical replicates, while biological replicates are displayed separately (n = 3 for untagged CUT&RUN, n = 2 for each tagged cell line). H. As in S5G but visualized over putative silencers (defined as gene-distal DHSs overlapping an H3K27me3 peak; K27me3 ChIP-seq from GSE123174, DNase-seq from GSM1014154). I. As in S5G but visualized over all gene-distal DHSs (DNase-seq from GSM1014154).

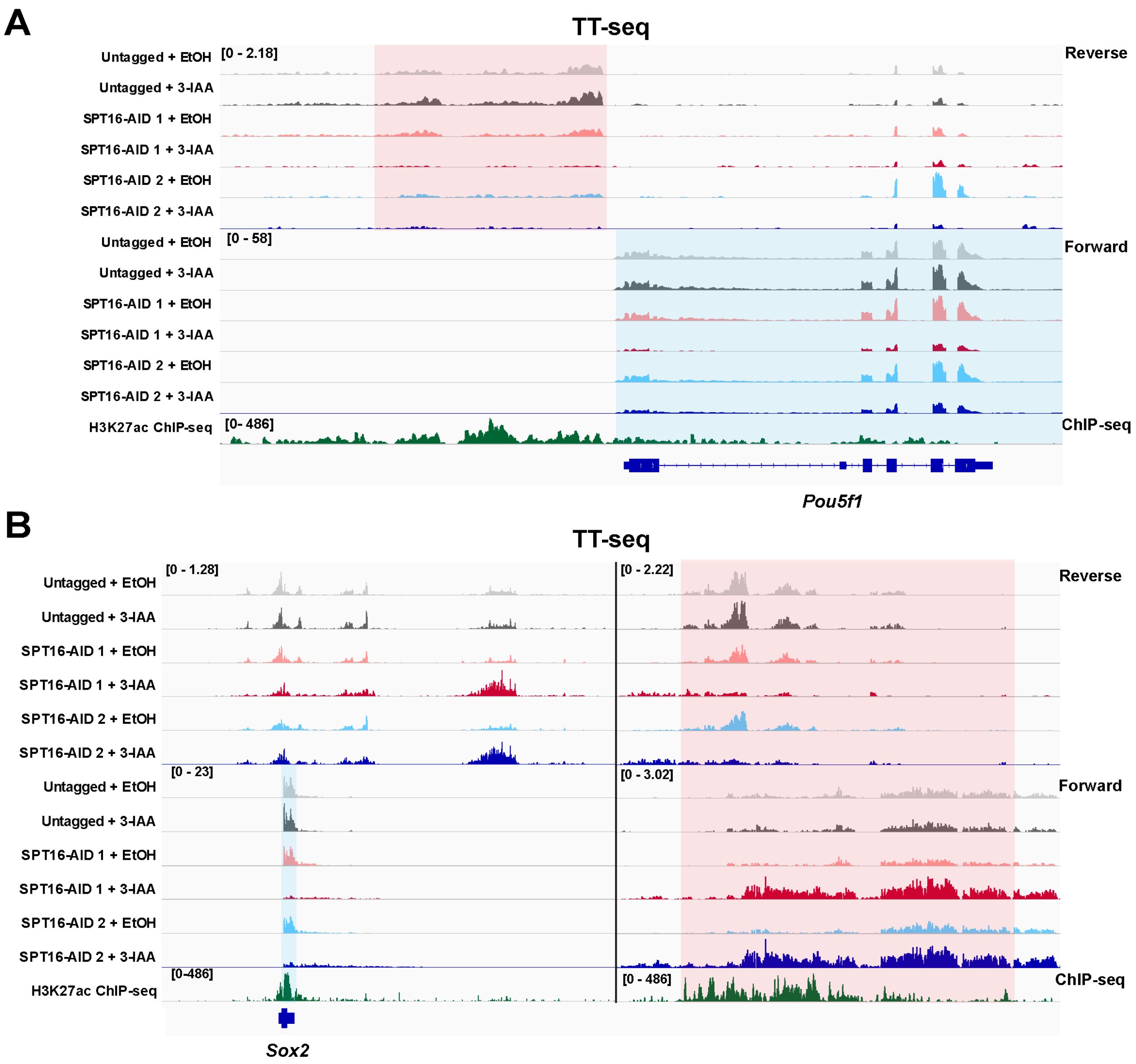

Fig. S6. FACT depletion reduces transcription at superenhancers. A. IGV genome browser tracks depicting nascent transcription at the *Pou5f1* locus, along with published H3K27ac ChIP-seq data. Red shaded area denotes a proximal superenhancer of *Pou5f1* transcription, while blue shaded area denotes the *Pou5f1* gene. Browser tracks represent merged technical replicates (n = 3), while biological replicates are displayed separately. B. IGV genome browser tracks depicting nascent transcription at the *Sox2* locus. Browser tracks represent merged technical replicates (n = 3), while biological replicates are displayed separately. Two individually scaled windows are

shown to highlight eRNA transcription from the *Sox2* distal superenhancer (red shaded area) and nascent transcription from the *Sox2* genomic locus (blue shaded area).

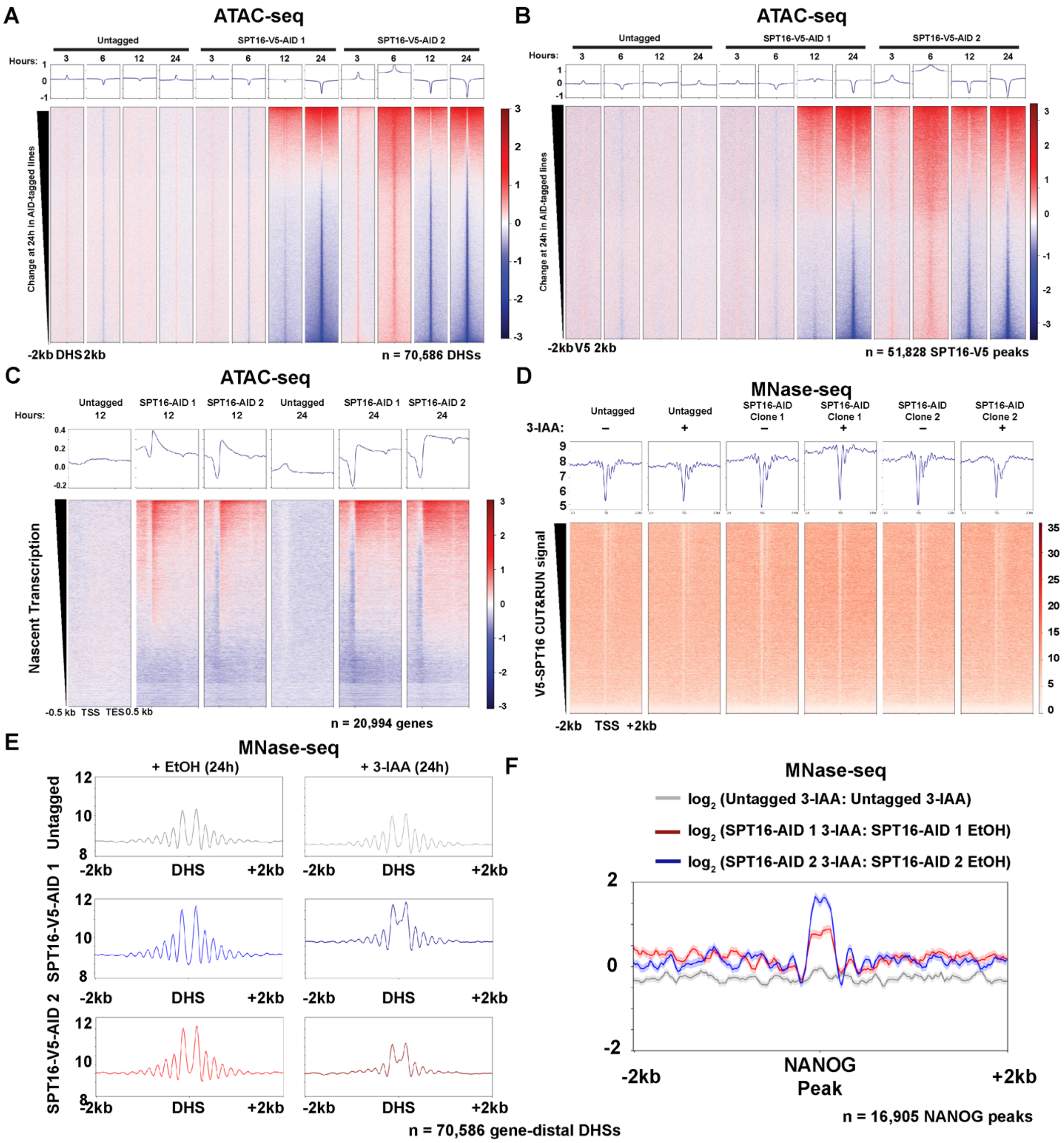

Fig. S7. FACT depletion disrupts nucleosome positioning and chromatin accessibility at regulatory regions. A-B. Timecourse of differential chromatin accessibility at gene-distal DHSs (A) and SPT16-V5 binding sites (B). Data are sorted by descending accessibility change in 24-

hour depletion samples (tagged lines only). Differential signal was calculated as in Fig. 6A. Per timepoint, n = 1 per tagged cell line, n = 2 per untagged cell line. Significance of altered chromatin accessibility at gene-distal DHSs (A) was analyzed by Friedman tests and corrected for multiple comparisons via Dunn's test ($p < 0.0001$ overall, adj. $p < 0.01$ for each individual comparison except between Untagged 3h and 12h experiments). Data were similarly analyzed over SPT16-V5 binding sites (B) ($p < 0.0001$ overall, adj. $p < 0.01$ for each individual comparison except between Untagged 3h and 12h experiments). C. Metagene plots depicting changes in chromatin accessibility over FACT-bound gene bodies after 12-24 hours of IAA treatment. Data are sorted by nascent transcription in control samples as in Fig. 2A. Differential signal was calculated as in Fig. 6. Data were analyzed as in panel A over whole genes ($p < 0.0001$ overall, adj. $p < 0.01$ for each individual comparison except between Untagged 0h, 3h, 12h, and 24h experiments). D. Heatmap depicting averaged nucleosome occupancy near RefSeq mRNA TSSs. Averaged replicates are shown as a single plot (n = 3 for untagged, n = 2 for each AID-tagged clone). Data were analyzed as in panel A over whole genes ($p < 0.0001$ overall, adj. $p < 0.0001$ for each individual comparison). E. Metaplots of MNase-seq data following 24 hours of SPT16 depletion, visualized over gene-distal DHSs. Metaplots shown represent merged technical replicates, while biological replicates are shown separately (n = 3 for untagged, n = 2 for each AID-tagged clone). MNase-seq data visualized over gene-distal DNaseI hypersensitive sites, ±2 kb (DNase-seq from GSM1014154). These data are presented as differential profiles in one metaplot in Fig. 6E. Shaded area represents standard error in either direction for each bin. Data were analyzed as in panel A over TSS-proximal regions ($p < 0.0001$ overall, adj. $p < 0.0001$ for each individual comparison). F. Metaplots of differential nucleosome occupancy between IAA and vehicle-treated samples, visualized over NANOG ChIP-seq binding sites, ±2 kb (ChIP-seq from GSE11724). Averaged replicates are shown as a single line (n = 3 for untagged, n = 2 for each AID-tagged clone). Tagged samples are shown in red and blue, while untagged samples are shown in grey.

Shaded area indicates standard error. Data were analyzed as in panel A over gene-distal DHSs ($p < 0.0001$ overall, adj. $p < 0.0001$ for each individual comparison).

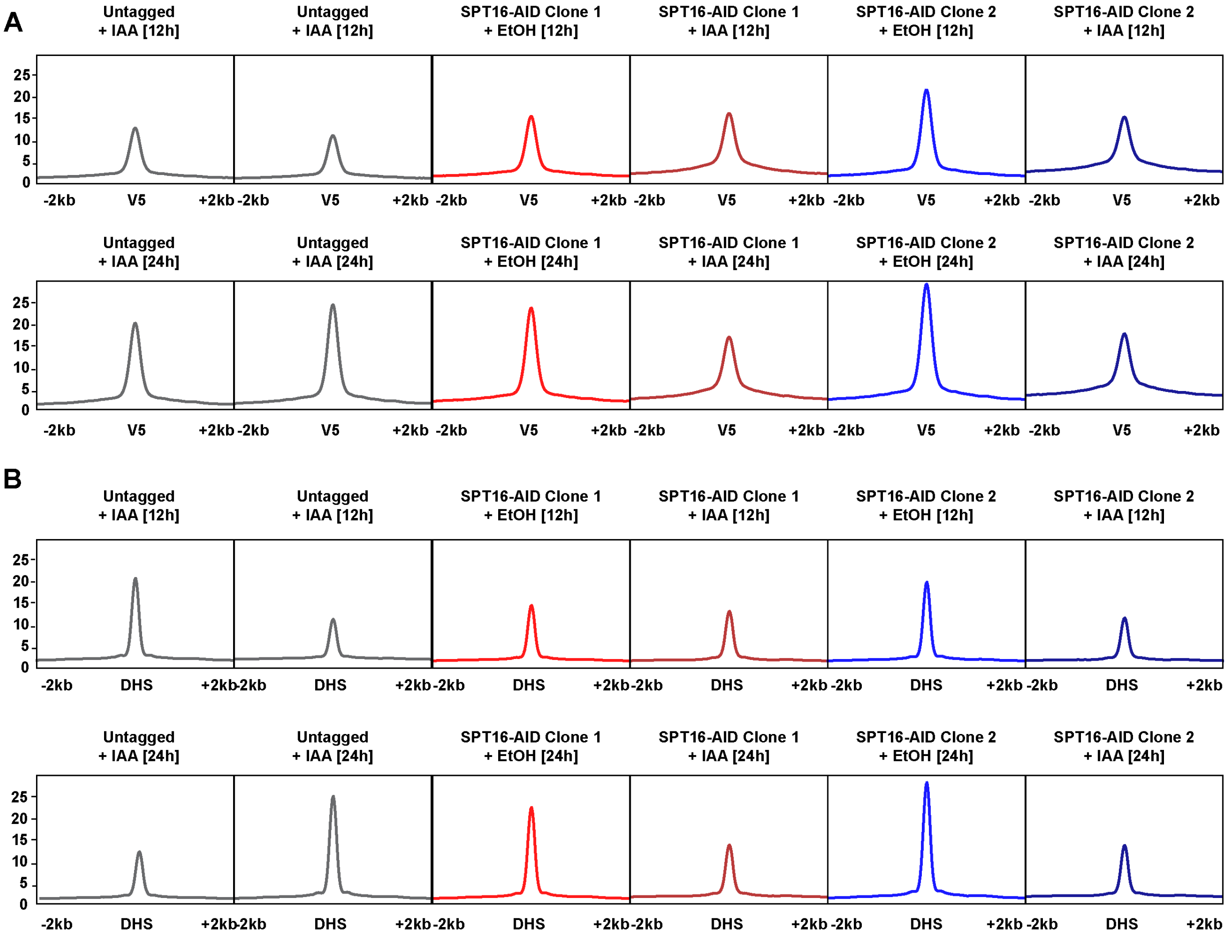

Fig. S8. FACT depletion disrupts chromatin accessibility at regulatory regions.

Normalized ATAC-seq signal plotted individually by cell line and treatment condition at SPT16-V5 binding sites (A), and gene-distal DHSs (B). Because changes in chromatin accessibility were minimal after less than 12-hour IAA treatment, only experiments following at least 12-hour treatment are shown. For experiments in untagged cells, n = 2 merged replicates, displayed as a single track; otherwise, n = 1 per timepoint. (DNase-seq from GSM1014154)

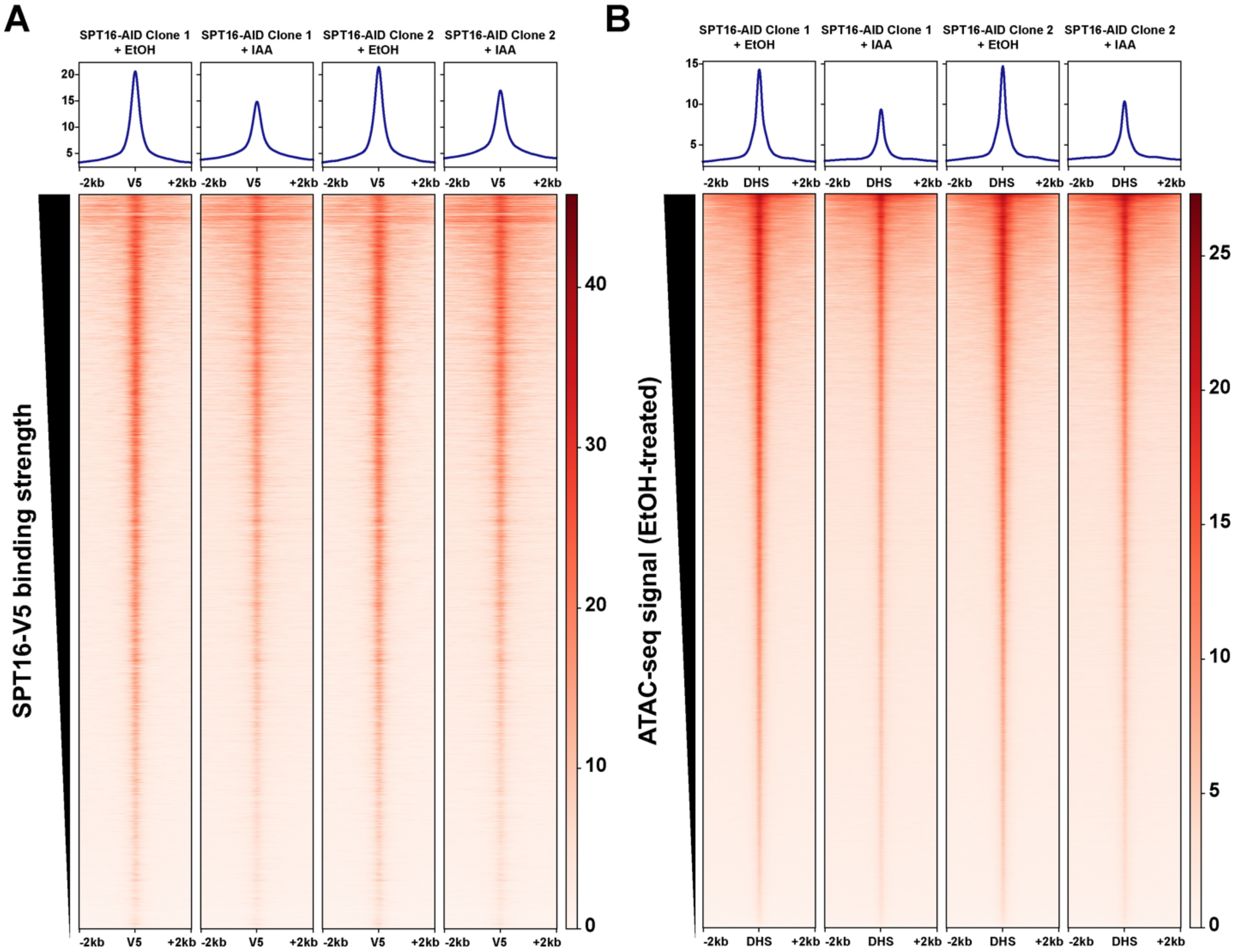

Fig. S9. Washout of IAA does not restore chromatin accessibility changes induced by 12-hour FACT depletion. Normalized ATAC-seq signal plotted individually by cell line and treatment condition at SPT16-V5 binding sites (A), and gene-distal DHSs (B). n = 2 merged replicates, displayed as a single track (DNase-seq from GSM1014154).

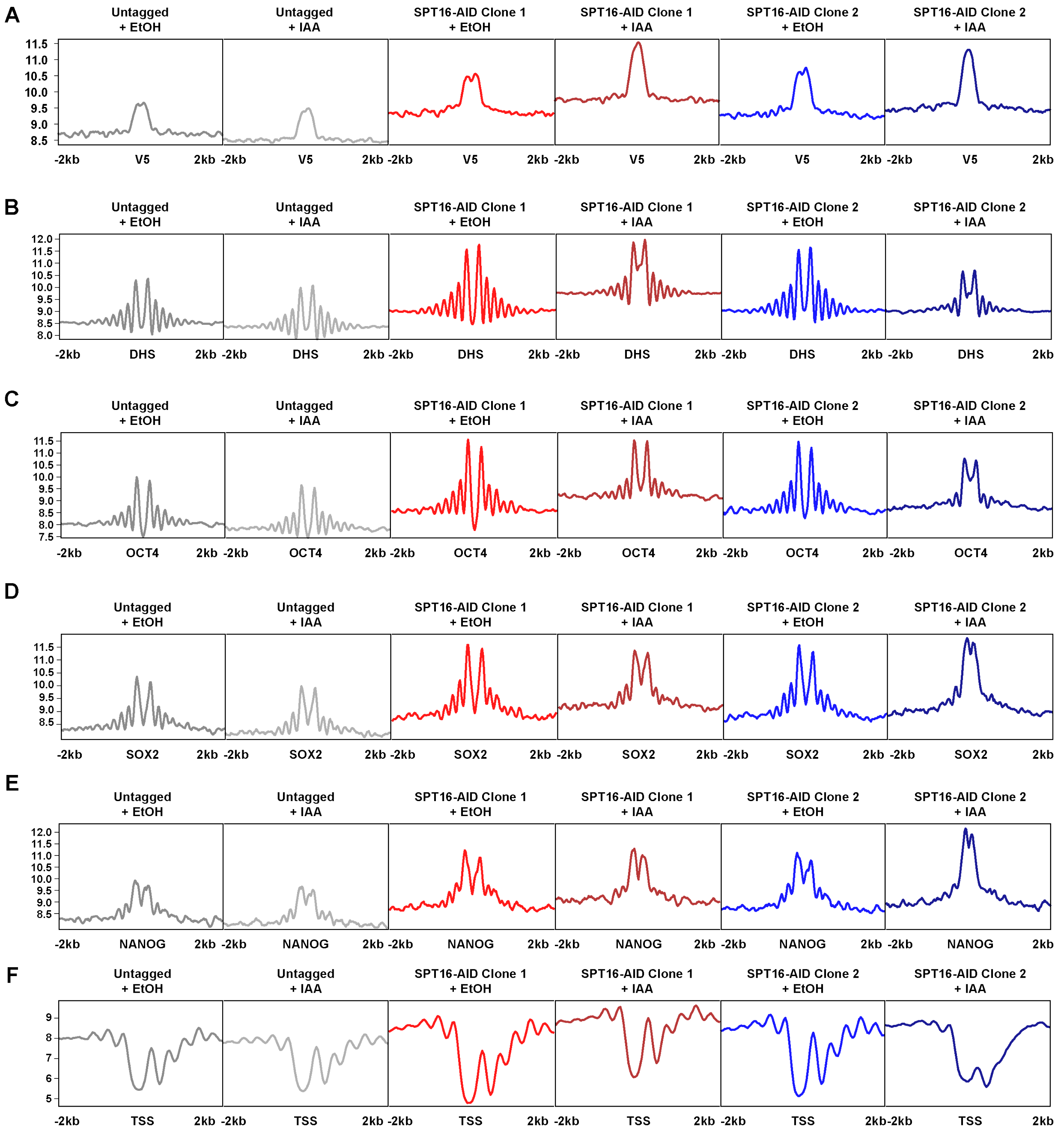

Fig. S10. FACT depletion disrupts nucleosome positioning at regulatory regions.

Normalized MNase-seq signal plotted individually by cell line and treatment condition at SPT16-V5 binding sites (A), gene-distal DHSs (B), ChIP-seq peaks for OCT4 (C), SOX2 (D), and NANOG (E), and annotated mm10 TSSs (F). For tagged lines, n = 2 merged replicates, displayed as a single track; for untagged lines, n = 3 merged replicates, displayed as a single track. (DNase-seq from GSM1014154; ChIP-seq from GSE11724)

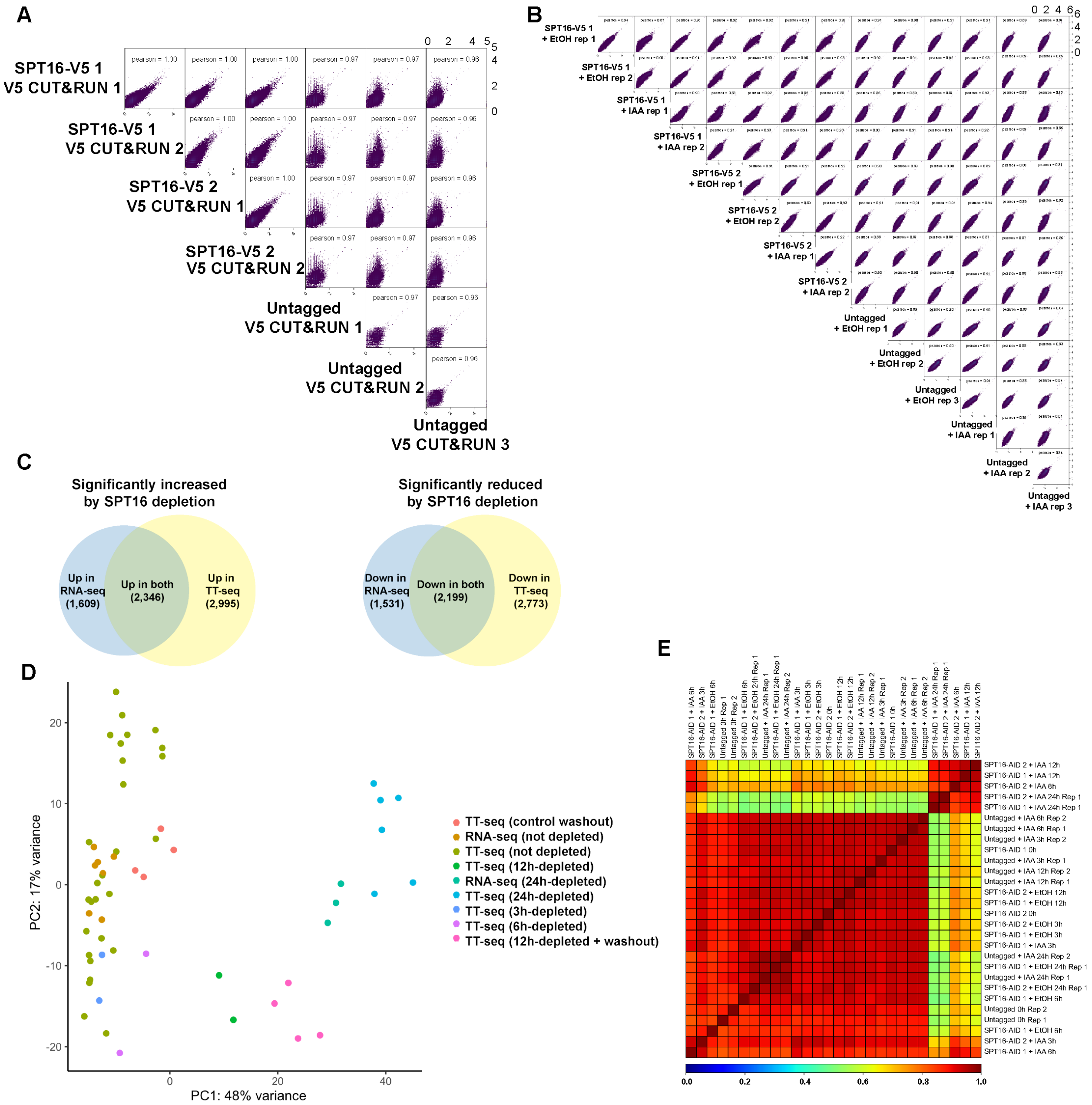

Fig. S11. Validation of NGS datasets. A. Pairwise scatterplot showing Pearson correlation between SPT16-V5 anti-V5 CUT&RUN and Untagged anti-V5 CUT&RUN. Individual technical replicates are compared for each sample. Bins represent average coverage over 5 kb regions of the genome. B. Pairwise scatterplot showing Pearson correlation between MNase-seq samples. Individual technical replicates are compared for each sample. Bins represent average coverage over 5 kb regions of the genome. C. Number of genes with transcripts significantly altered after

24-hour IAA treatment and subsequent RNA-seq or TT-seq. Only genes with a DESeq2 adjusted p-value < 0.05 were included, and only genes with log_2 fold changes in the same direction after IAA treatment were marked as overlapping between techniques. D. PCA plot depicting relationships between all transcriptomic samples. E. Pairwise heatmap depicting Pearson correlation between ATAC-seq samples over all consensus ATAC-seq peaks called via PEPATAC (those existing in over half of the total datasets).

REAGENT or RESOURCE	SOURCE	IDENTIFIER
**Antibodies**		
Mouse anti-V5 monoclonal antibody	Invitrogen	Cat: R906-25; RRID: AB_2556564; lot 1923773
Mouse anti-SSRP1 monoclonal antibody	BioLegend	Cat: 609702; RRID: AB_315731; lot B280320
Mouse anti-Beta-actin monoclonal antibody	Sigma	Cat: A1978; RRID: AB_476692; lot 037M4782V
Rabbit anti-SPT16 monoclonal antibody	Cell Signaling	Cat: 12191S; RRID: AB_2732025; lot 1
Goat anti-mouse polyclonal antibody	BioRad	Cat: 170-6516; RRID: AB_11125338; lot 64147779
Goat anti-rabbit polyclonal antibody	BioRad	Cat: 170-6515; RRID: AB_11125142; lot 64149722
Rabbit anti-OCT4 polyclonal antibody	Stemgent (ReproCELL Technologies)	Cat: 09-0023; RRID: AB_2167689; lot J17070000000001
Goat anti-rabbit fluorophore-conjugated antibody	Vector Labs	Cat: DI-1488; RRID: AB_2336402; lot ZJ0214
**Chemicals, peptides, and recombinant proteins**		
Protein A/Protein G/MNase fusion protein	Addgene	https://www.addgene.org/123461/; RRID: Addgene_123461
IAA	Sigma	Cat: I3750
4-Thiouridine	Carbosynth	T4509
Biological samples		
pX330 plasmid	Addgene	http://www.addgene.org/42230/; RRID: Addgene_42230
pAG/MNase plasmid	Addgene	https://www.addgene.org/123461/; RRID: Addgene_123461
Critical commercial assays		
Vector Red Alkaline Phosphatase staining kit	Vector Laboratories	RRID: AB_2336847
ZeroBlunt TOPO PCR cloning kit	Invitrogen	Cat: 451245
**Deposited data**		
SPT16-V5 CUT&RUN data	This study	GSE181624
FACT depletion MNase-seq	This study	GSE181624
SPT16 depletion ATAC -seq data	This study	GSE181624
SPT16 depletion TT-seq data	This study	GSE181624
SPT16 depletion RNA-seq data	This study	GSE181624
OCT4, SOX2, and NANOG ChIP-seq data	Marson *et al.*, 2008	GSE11724
DNaseI Hypersensitive sites	ENCODE DCC	GSM1014154
H3K4me3, H3K27ac ChIP-seq	ENCODE DCC	GSE32218
H3K4me1, H3K36me3 ChIP-seq	ENCODE DCC	GSE31039
H3K27me3 ChIP-seq	Mu *et al.*, 2018	GSE123174
**Experimental models: Cell lines**		
E14 ES cell line	Hooper *et al.*, 1987	RRID: CVCL_C320
E14 TG2a CAG-Tir1-puro (Rosa26-CAG-nlsTir1-IRES-puro)	Baker *et al.*, 2016	

E14 TG2a CAG-Tir1-puro (Rosa26-CAG-nlsTir1-IRES-puro), with SPT16-V5-AID, clone 1	This study	
E14 TG2a CAG-Tir1-puro (Rosa26-CAG-nlsTir1-IRES-puro), with SPT16-V5-AID, clone 2	This study	
**Oligonucleotides**		
Pou5f1 (OCT4) RT-qPCR primer; F TGGAGGAAGCCGACAACAATGAGA	Frum et al., 2013	
Pou5f1 (OCT4) RT-qPCR primer; R TGGCGATGTGAGTGATCTGCTGTA	Frum *et al.*, 2013	
Pgk1 RT-qPCR primer; F GGGTGGATGCTCTCAGCAAT	Panina et al., 2018	
Pgk1 RT-qPCR primer; R GTTCCTGGTGCCACATCTCA	Panina *et al.*, 2018	
Supt16 RT-qPCR primer; F ACTACCGGCGAGTGAAGAGA	This study	
Supt16 RT-qPCR primer; R CAACACCCACCGATACAACA	This study	
Ssrp1 RT-qPCR primer; F CAGAGACATTGGAGTTCAACGA	This study	
Ssrp1 RT-qPCR primer; R GCCCGTCTTGCTGTTCTTAAAG	This study	
Nanog RT-qPCR primer; F ATGAAGTGCAAGCGGTGGCAGAAA	Li et al., 2013	
Nanog RT-qPCR primer; R CCTGGTGGAGTCACAGAGTAGTTC	Li *et al.*, 2013	
Sox2 RT-qPCR primer; F TTTTCTAGTCGGCATCACCG	Zhang et al., 2016	
Sox2 RT-qPCR primer; R ACAAGAGAATTGGGAGGGGT	Zhang *et al.*, 2016	
Supt16 C-terminal outside check primer; F GAAGGTGCAGAGCAGTTGAGC	This study	
Supt16 C-terminal inside check primer; R AGCTTGGTCCGCACAAATGG	This study	
Supt16 C-terminal inside check primer; F CCTCTGCCTTCCAAGTGCTG	This study	
Supt16 C-terminal pX330 cloning primer; F caccgTGGAACCACGGTTAGAGCCA	This study	
Supt16 C-terminal pX330 cloning primer; R aaacTGGCTCTAACCGTGGTTCCAc	This study	

Table S2. Key reagents, cell lines, and datasets used in this work.
