# Supplementary material for: FACT regulates pluripotency through proximal and distal regulation of gene expression in murine embryonic stem cells
Source: BMC Biol. 2023 Aug 4;21:167. doi: 10.1186/s12915-023-01669-0 (PMC10403911; doi:10.1186/s12915-023-01669-0)

Figure 1B (V5-SPT16)

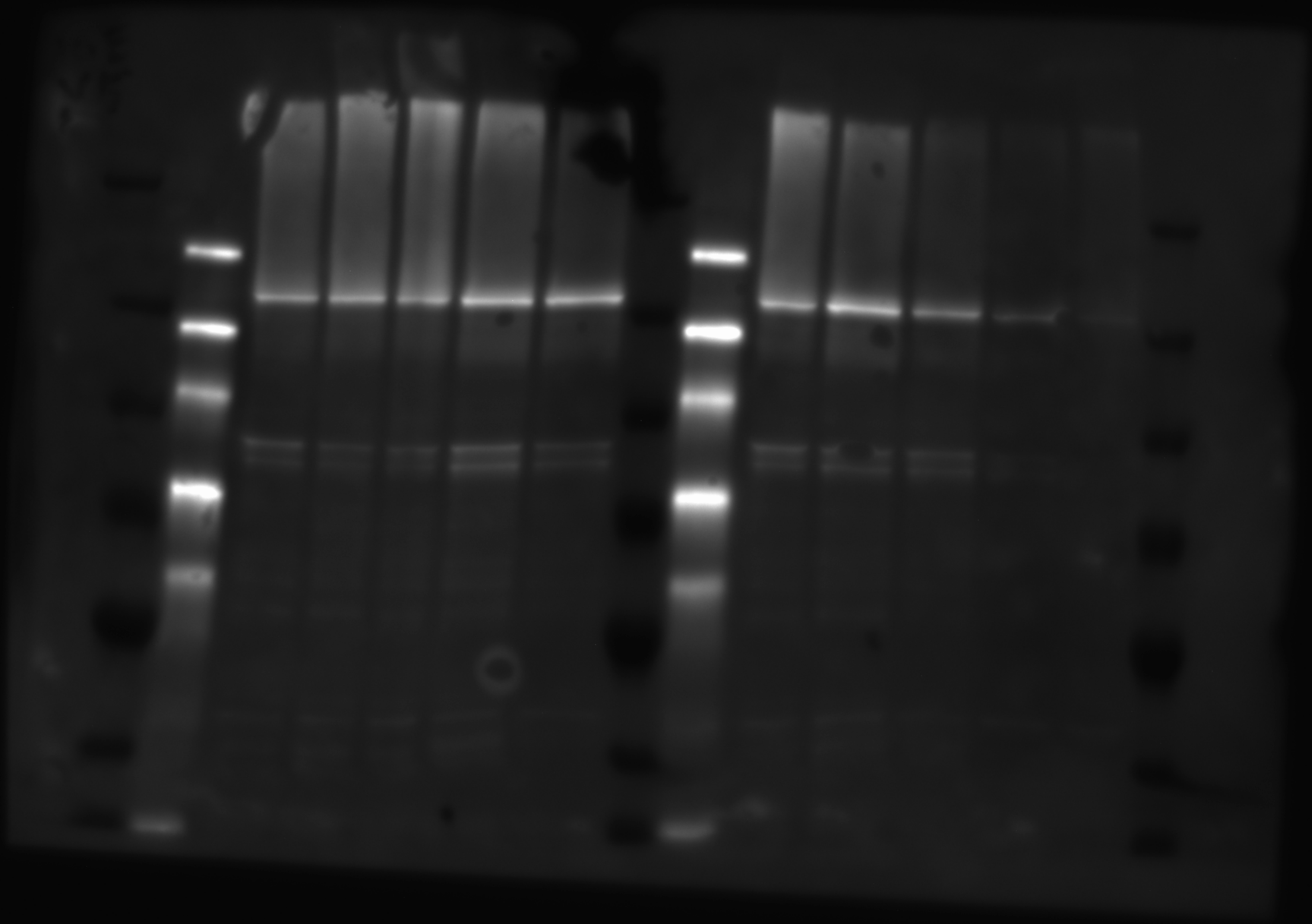

Jul/13/2021 12:06:47

Figure 1B (Actin)

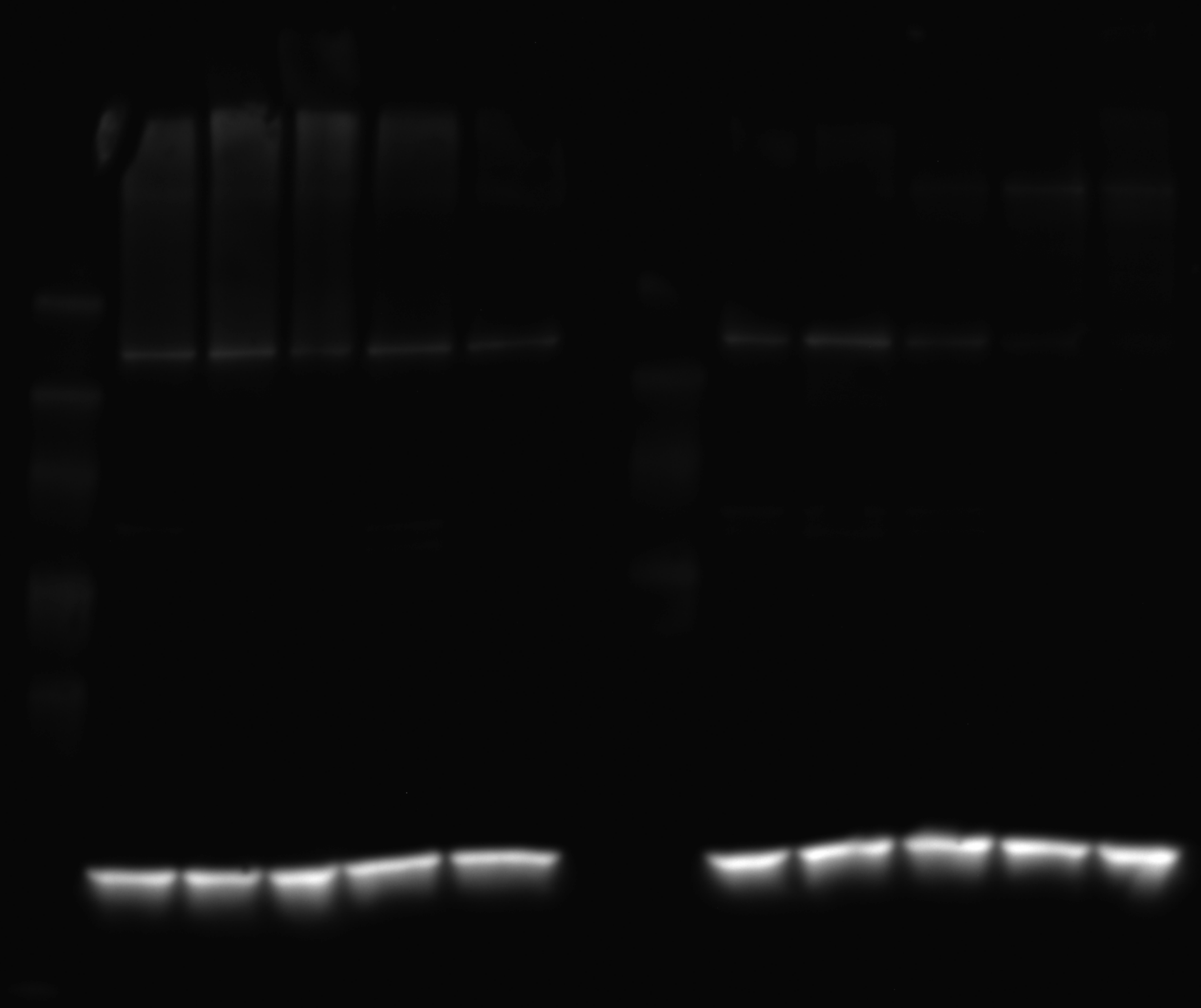

Jul/14/2021 12:16:34

Figure S1A (V5-SPT16)

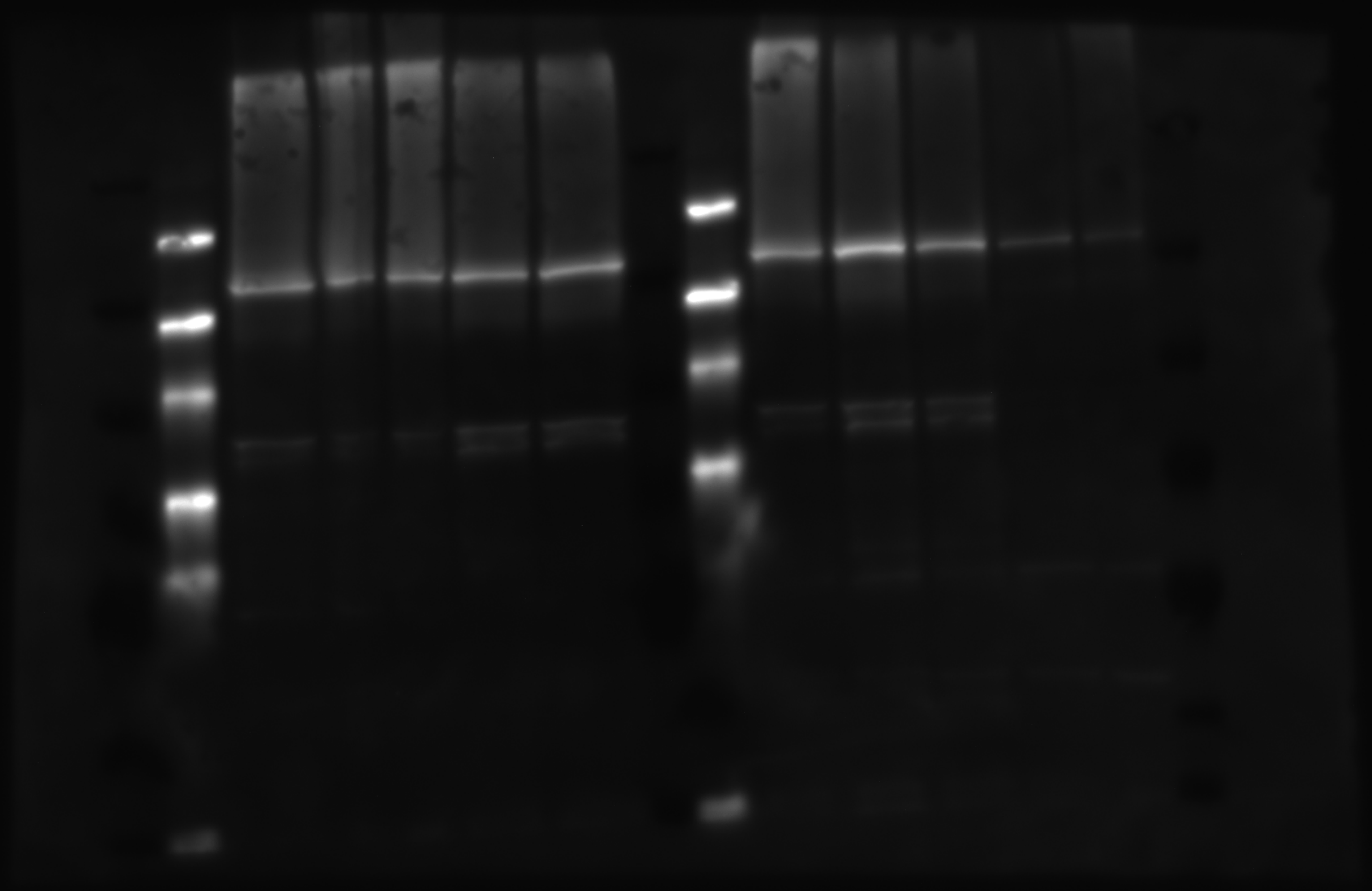

Figure S1A (Actin)

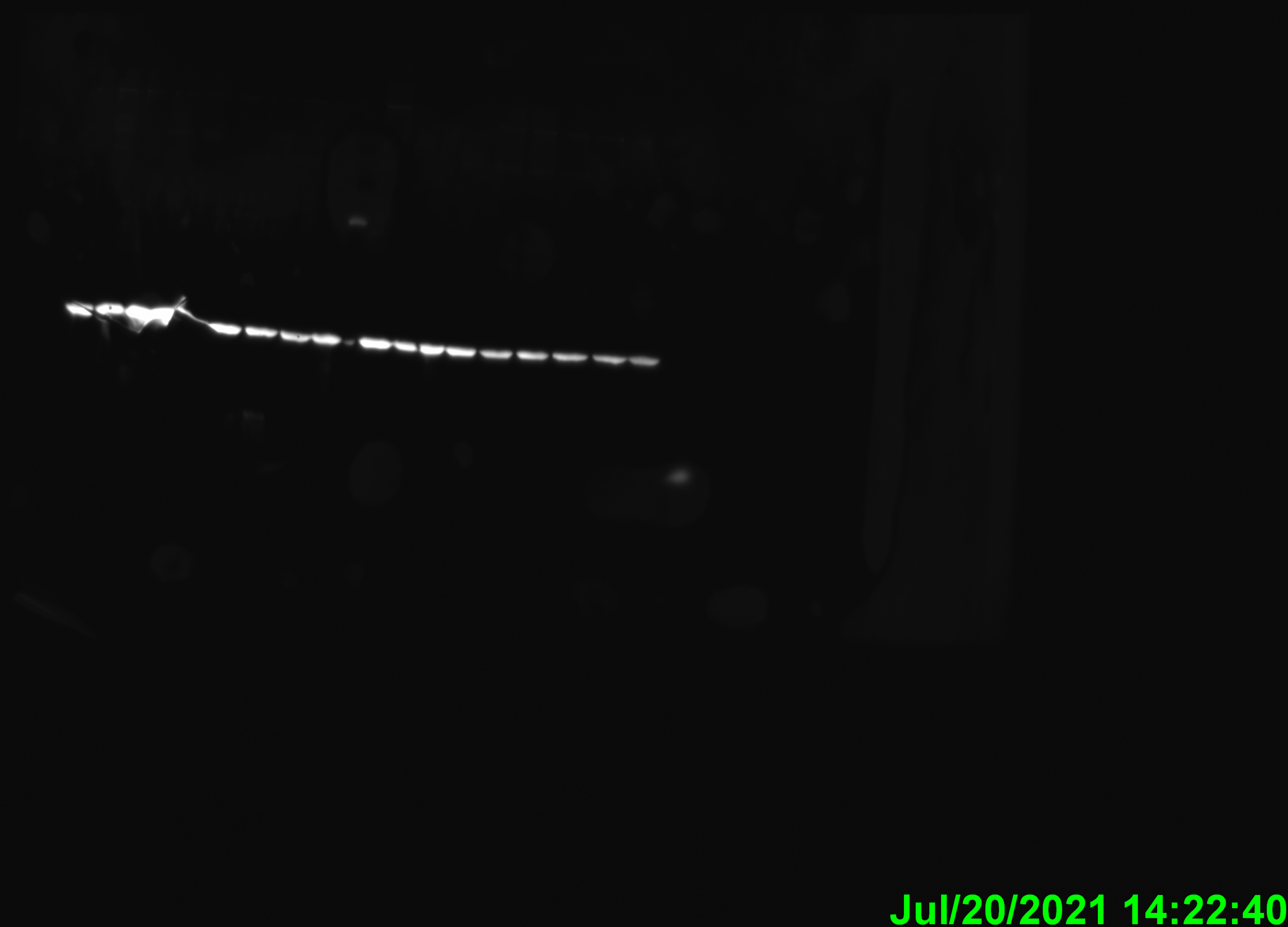

Figure S1B (OCT4)

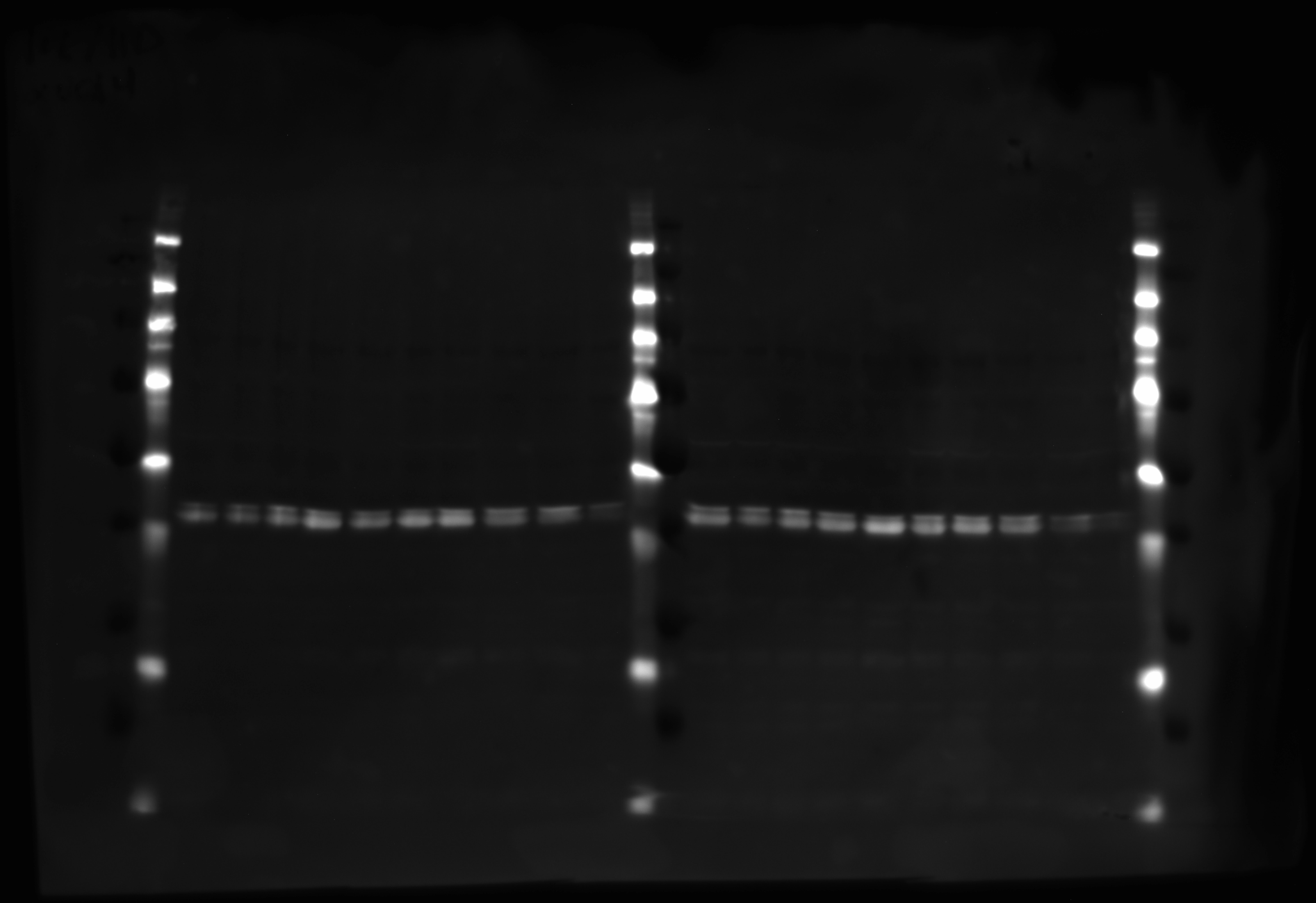

Jul/16/2021 12:59:52

Figure S1B (SSRP1 [top] and Actin [bottom])

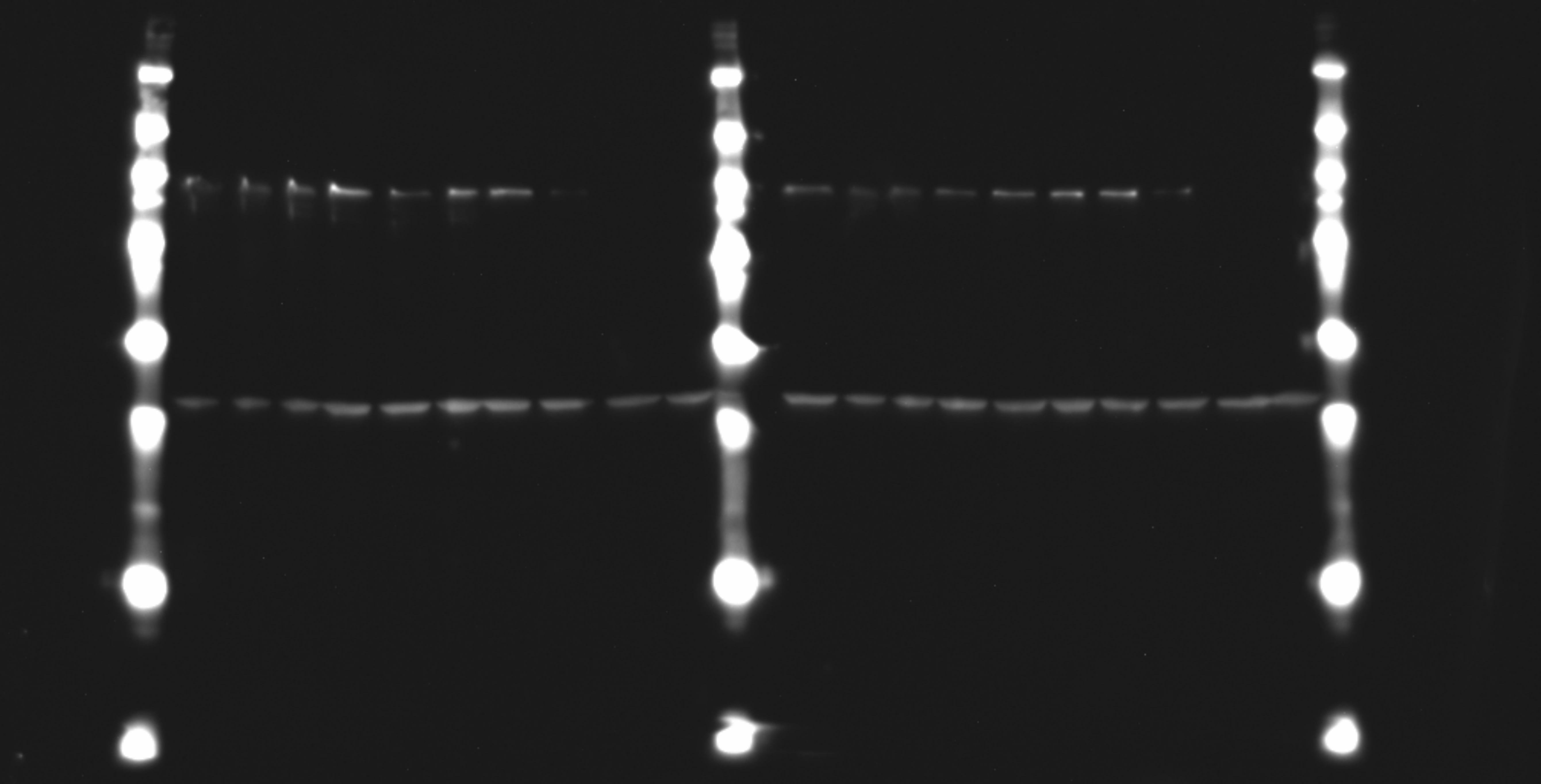

Jul/19/2021 12:22:02

Figure S1C (V5-SPT16)

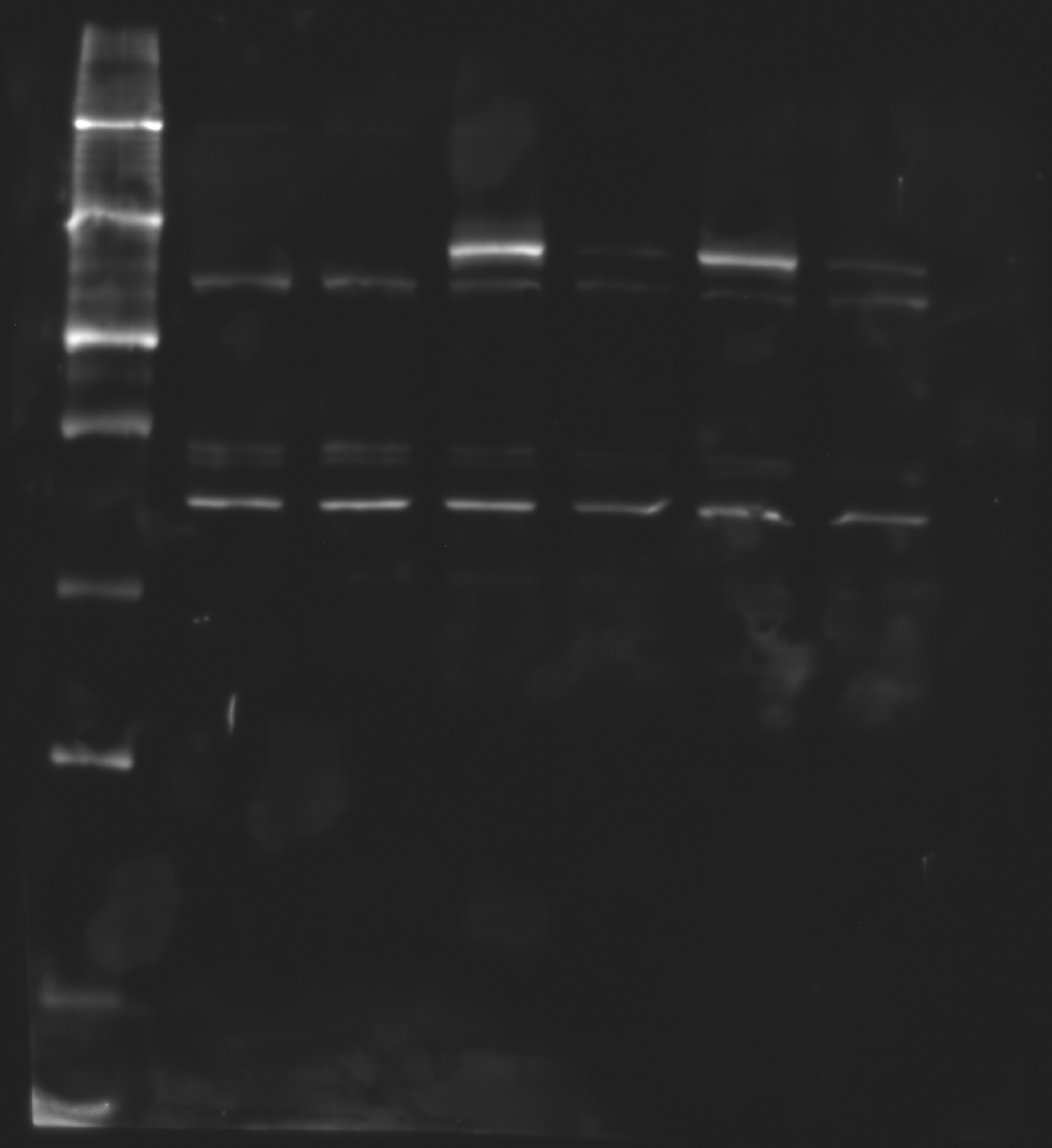

Figure S1C (SSRP1)

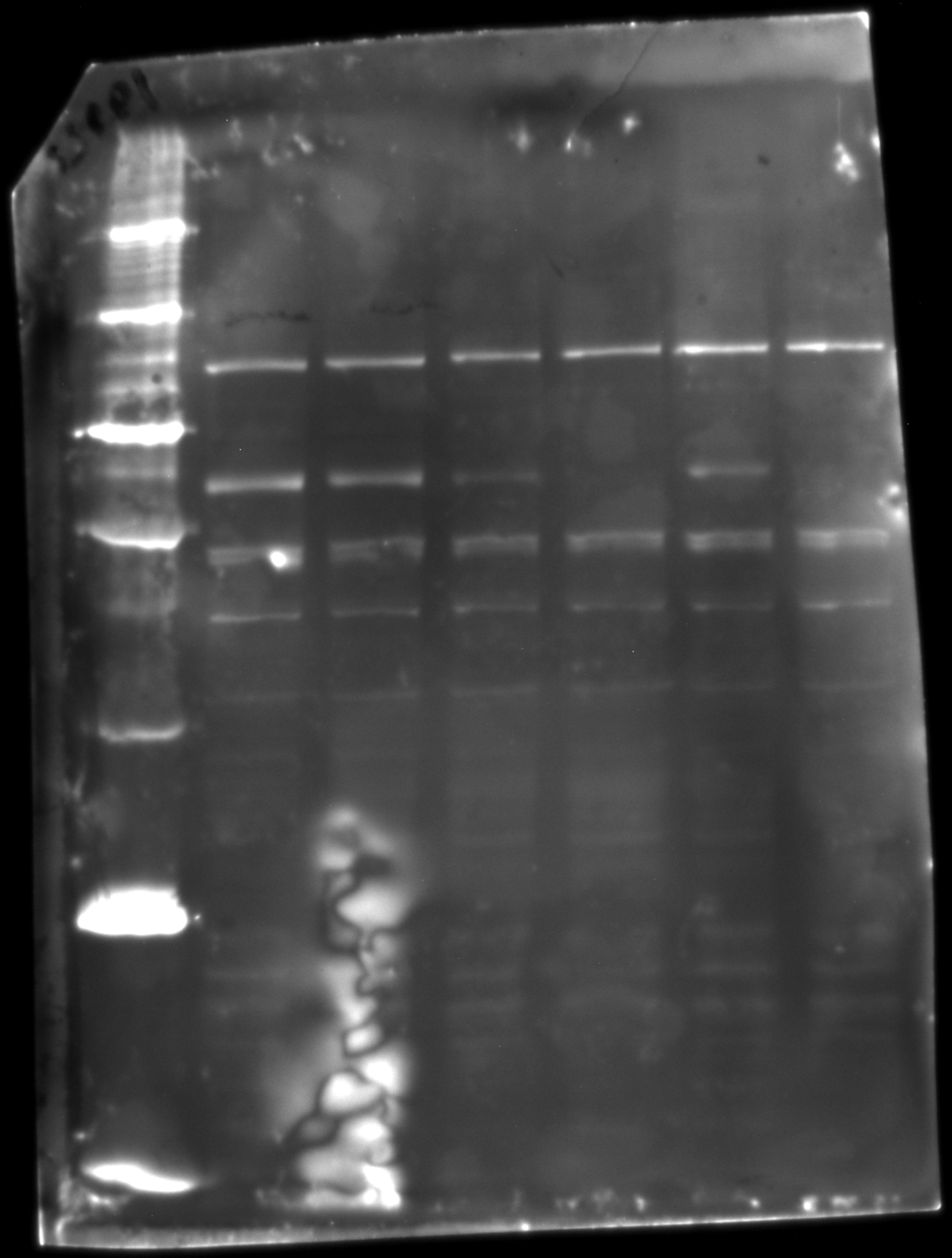

Figure S1C (Actin)

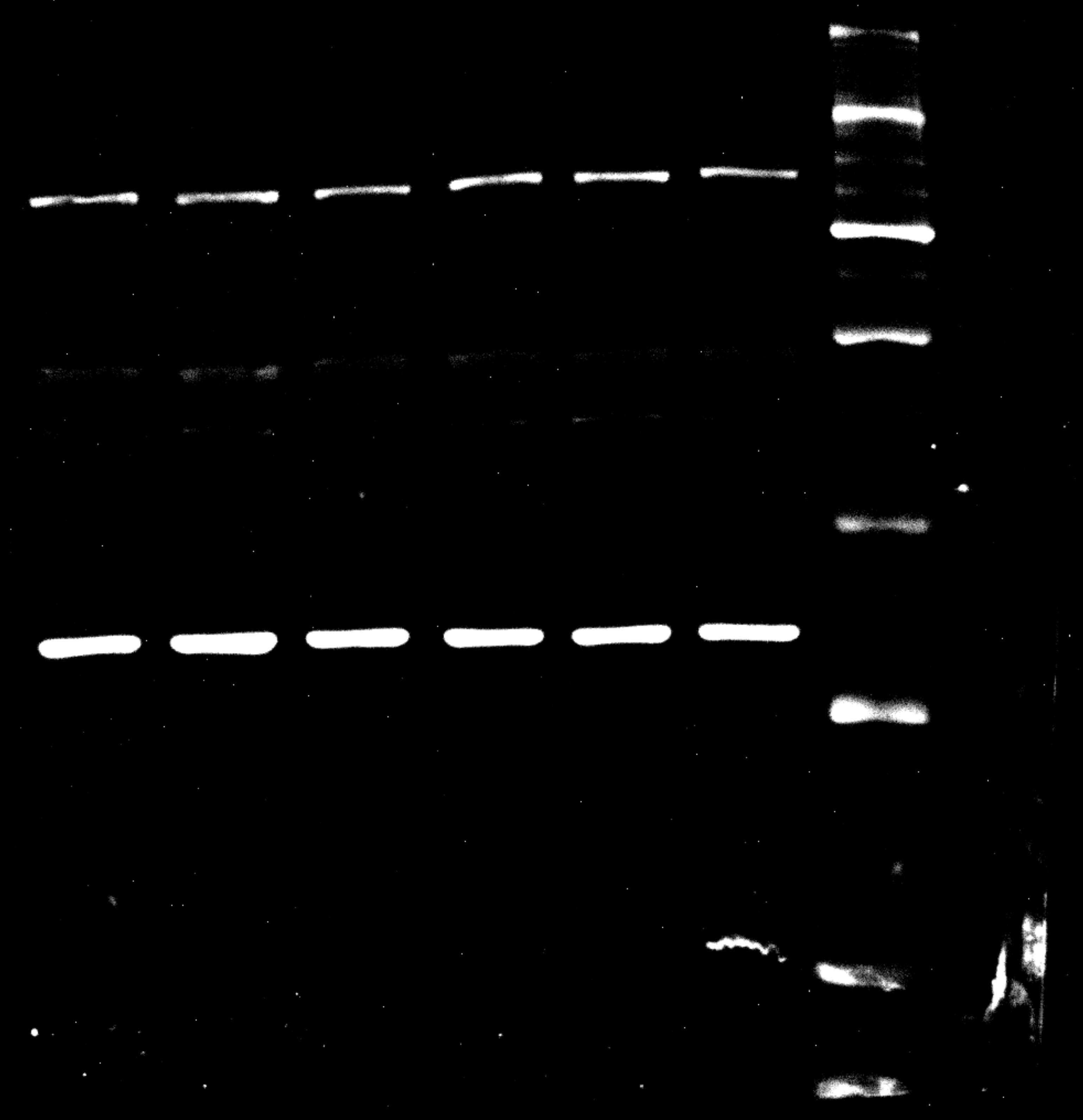

Supplement: Supplementary file 3 — Additional file 3. Unedited, uncropped Western blots displayed in Figs. 1 and S1. Blots are presented in the following order: Fig. 1A (V5-SPT16); Fig. 1A (Actin); Additional File 1: Fig. S1A (V5-SPT16); Additional File 1: Fig. S1A (Actin); Additional File 1: Fig. S1B (OCT4); Additional File 1: Fig. S1B (SSRP1 and Actin); Additional File 1: Fig. S1C (V5-SPT16); Additional File 1: Fig. S1C (SSRP1); Additional File 1: Fig. S1C (Actin). [file 12915_2023_1669_MOESM3_ESM.pdf]
